# A Reasonable Officer: Examining the Relationships Among Stress, Training, and Performance in a Highly Realistic Lethal Force Scenario

**DOI:** 10.3389/fpsyg.2021.759132

**Published:** 2022-01-17

**Authors:** Simon Baldwin, Craig Bennell, Brittany Blaskovits, Andrew Brown, Bryce Jenkins, Chris Lawrence, Heather McGale, Tori Semple, Judith P. Andersen

**Affiliations:** ^1^Department of Psychology, Carleton University, Ottawa, ON, Canada; ^2^Royal Canadian Mounted Police, Ottawa, ON, Canada; ^3^Police Research Lab, Carleton University, Ottawa, ON, Canada; ^4^Department of Psychology, University of Toronto Mississauga, Mississauga, ON, Canada

**Keywords:** police, stress, training, use of force, objective reasonableness standard

## Abstract

Under conditions of physiological stress, officers are sometimes required to make split-second life-or-death decisions, where deficits in performance can have tragic outcomes, including serious injury or death and strained police–community relations. The current study assessed the performance of 122 active-duty police officers during a realistic lethal force scenario to examine whether performance was affected by the officer’s level of operational skills training, years of police service, and stress reactivity. Results demonstrated that the scenario produced elevated heart rates (i.e., 150 beats per minute), as well as perceptual and cognitive distortions, such as tunnel vision, commensurate with those observed in naturalistic use of force encounters. The average performance rating from the scenario was 59%, with 27% of participants making at least one lethal force error. Elevated stress reactivity was a predictor of poorer performance and increased lethal force errors. Level of training and years of police service had differential and complex effects on both performance and lethal force errors. Our results illustrate the need to critically reflect on police training practices and continue to make evidence-based improvements to training. The findings also highlight that while training may significantly improve outcomes, flawless performance is likely not probable, given the limits of human performance under stress. Implications for the objective reasonableness standard, which is used to assess the appropriateness of force in courts of law, are discussed.

## Introduction

Police officers encounter critical incidents that have the hallmark characteristics of a situation that would cause a physiological stress response: namely – they are unpredictable, potentially uncontrollable, novel, and often involve time pressure (Sapolsky, 2004; Alison and Crego, 2012; Violanti, 2014). Under these circumstances, officers are occasionally required to make life-or-death decisions, often in a split-second, to preserve and protect the lives of both the public and themselves (Artwohl, 2002). Video footage of certain police–public encounters highlights deficits in officer performance, including errors in the decision to use lethal force. Such incidents can have tragic consequences, including serious injury or death and strained police–community relations. Occasionally, such incidents can also lead to the incarceration of police officers and legal liability for law enforcement agencies (LEAs) that have not adequately prepared their officers for critical incidents (e.g., Public Prosecution Service of Canada, 2018).

Existing research and theoretical knowledge indicate that stress can adversely impact performance, but that training and experience can moderate stress reactivity and improve performance through the appraisal process (e.g., Driskell and Salas, 1996). Thus, using a sample of Canadian police officers, the aim of the current study is to examine the level of performance that can reasonably be expected under conditions that elicit high levels of stress, based on officers’ years of experience and the level of training they have received. Critical reflection on training practices and evidence-based improvements to training may be called for if systemic errors or deficiencies in performance are observed in realistic scenarios. Results from this study may also provide new evidence to inform the objective reasonableness standard, which is used to assess the appropriateness of force in courts of law (Cyr, 2016; Zamoff, 2020).

### Use of Force and the Objective Reasonableness Standard

The authority for police to use force in Canada is granted under section 25 of the Criminal Code (1985), whereby police officers who are acting on reasonable grounds are authorized to use as much force as necessary to enforce the law. In the case of *R. v. Nasogaluak* (McLachlin et al., 2010, p. 208), the Supreme Court of Canada further established that the “allowable degree of force is constrained by the principles of proportionality, necessity and reasonableness.” Where lethal force is concerned, the force must also be necessary for the purpose of self-preservation or the protection of others from death or grievous bodily harm (Criminal Code, 1985). To assess the appropriateness of an officer’s use of force, several guiding principles from international case law have become entrenched in the Canadian criminal justice system. Foremost is the U.S. Supreme Court case of *Graham v. Connor* (Rehnquist, 1989, p. 387), which established the objective reasonableness standard, instructing that the “…‘reasonableness’ of a particular use of force must be judged from the perspective of a reasonable officer on the scene, and its calculus must embody an allowance for the fact that police officers are often forced to make split-second decisions about the amount of force necessary in a particular situation.” In essence, given the totality of the circumstances known at the time and without hindsight bias, would other reasonably prudent officers respond in the same or similar way (Alpert and Smith, 1994; International Association of Chiefs of Police, 2020)?

Scholars have provided research evidence of neurophysiological factors (e.g., cognitive and perceptual distortions) that might frame the perceptions and actions of a reasonable officer on the scene (e.g., Klinger and Brunson, 2009). However, the use of such research in court may be the exception rather than the rule (DuCharme, 2002). Indeed, critics argue that the objective reasonableness standard lacks an evidence-based foundation and that assessments of reasonableness focus too much on the general dangers and stressful nature of policing (Fagan and Campbell, 2020; Zamoff, 2020). To remedy this, Zamoff (2020) recently proposed that in determining the perspective of a reasonable officer, the courts should more heavily weigh the officer’s experience and training, as well as the extent to which they adhered to or deviated from their training and the agency’s policies. While valuable, this approach also lacks evidence of the extent that these factors are related to performance and errors or are influenced by stress (Engel and Smith, 2009).

### Psychophysiological Threat Response

When presented with a threat, whether real or perceived, the body implicitly (i.e., below conscious awareness) engages in a series of physiological processes, colloquially known as the “fight-or-flight” response (Thayer and Sternberg, 2006; LeDoux and Pine, 2016). This evolutionary adaptive response promotes survival by immediately preparing the body’s physiological and cognitive capacities to meet the demands of the situation, while suppressing unnecessary functions, such as reproduction and digestion (Kemeny, 2003; Artwohl, 2008; Anderson et al., 2019). During the fight-or-flight response, the sympatho-adrenal response is triggered, which leads to a wide-spread release of catecholamines and hormones to power the survival response (McEwen, 1998; Lovallo, 2016). Specifically, the hypothalamic–pituitary–adrenal (HPA) axis is activated, which results in the rapid release of epinephrine (i.e., adrenaline) and cortisol (De Kloet et al., 1998; Lovallo, 2016). Cortisol increases blood sugar and prepares the body for energy expenditure by stimulating glucose production and mobilizing fatty acids (Lovallo, 2016; Tsigos et al., 2020). Concurrently, the autonomic nervous system (ANS) is engaged, stimulating the sympathetic nervous system (SNS) and suppressing the parasympathetic nervous system (PNS), which is associated with modifying the sympathetic response when necessary (e.g., focused attention) and performing “rest and digest” (i.e., recovery and repair) functions (Berntson and Cacioppo, 2004; Fridman et al., 2019). When the SNS is activated, stress hormones such as norepinephrine and epinephrine are released (Lovallo, 2016).

The cascade of these catecholamines, hormones, and glucose in the bloodstream from the stress system response stimulates increased heart rate (HR), blood pressure, and respiration (Tsigos and Chrousos, 2002; Chrousos, 2009). The rapid rise in energy, oxygenation, and blood flow is directed in greatest concentration to the heart, brain, and large muscles, while they are inhibited to other areas not required to respond to a threat, such as the digestive system (Tsigos and Chrousos, 2002). Therefore, activation of this sympatho-adrenal stress response improves chances of survival in the short term, by increasing resistance, strength, and focused attention (Tsigos and Chrousos, 2002; Artwohl, 2008; Fenici et al., 2011).

While fight-or-flight is an automatic behavioral and physiological response that is engaged without the need for higher-order cognitive processing, it can be sustained and moderated through psychological processes, such as threat appraisal, fear, and anxiety (Thayer and Sternberg, 2006; LeDoux and Pine, 2016; Chan and Andersen, 2020). The degree of SNS arousal depends primarily on the type of threat encountered and one’s perception of how severe it is (Kalisch et al., 2015; LeDoux and Pine, 2016). For example, when the threat of harm during an encounter with a subject is perceived by the officer as outweighing their ability to cope with the situation (e.g., based on experience and training), then the subject may continue to be appraised as a threat, maintaining the intensity of the emotional and physiological response (Folkman et al., 1986; Driskell and Salas, 1996; Anshel et al., 1997). While there is significant evidence that the body implicitly responds to a threat, what is less clear is the extent to which this response varies as a function of experience and training, as well as the impact it has on various aspects of performance.

### The Impact of Stress on Police Performance

Studies demonstrate that the impact of SNS arousal on performance is complex. The type of threat stimulus encountered, and the strength of the resulting threat response can improve or impair perceptual, cognitive, and motor performance depending on context (Arble et al., 2019; Bertilsson et al., 2019). Adaptive SNS arousal, which meets the demands of the situation, can be beneficial to performance (Yerkes and Dodson, 1908), such as shooting accuracy (e.g., Vickers and Williams, 2007), and threat-related decision-making (e.g., Akinola and Mendes, 2012). However, maladaptive stress arousal (i.e., too much or too little) is considered one of the main causes of human performance failure (Vine et al., 2016) and can result in degradation of task accuracy and increased task errors (Driskell and Salas, 1996; Nieuwenhuys et al., 2012). Growing evidence also suggests that performance deficits are related to both maladaptive SNS arousal and the suppression of the stress modulating parasympathetic influence (Saus et al., 2006; Andersen et al., 2018; Spangler et al., 2018). For example, impairments to response inhibition, resulting in more lethal force errors, can occur when the PNS is suppressed (Spangler et al., 2018).

Generally, stress-induced deficits primarily affect cognitive functions, such as perception, attention, and decision-making (Driskell and Salas, 1996; Di Nota et al., 2020). However, motor performance, in particular fine motor skills, is also affected (Staal, 2004; Nieuwenhuys and Oudejans, 2011a; Anderson et al., 2019). Since manipulating stressful real-world encounters for research purposes is unethical (Giessing et al., 2019), results from realistic scenario-based experiments form much of the existing knowledge about the impact of acute stress on performance among police officers. To date, this literature has revealed that stress inducing scenarios result in impairments to various aspects of police performance including shooting accuracy (Nieuwenhuys and Oudejans, 2010; Taverniers and De Boeck, 2014; Landman et al., 2016a), quality of skill execution (Renden et al., 2014; Nieuwenhuys et al., 2016), proportionality of force applied (Nieuwenhuys et al., 2012; Renden et al., 2017), and memory (Hope et al., 2016). The stress response also appears to have differential effects, whereby rehearsed and automated skills are influenced to a lesser degree (Vickers and Lewinski, 2012; Renden et al., 2017; Arble et al., 2019). These findings from experimental research with simulations are extremely important to draw conclusions about what *might* reasonably happen to performance in real-world stressful encounters (Giessing et al., 2019).

While few real-world studies exist, examinations of officer-involved shootings (OIS) have also uncovered stress-induced performance issues. For example, average hit rates ranging from 14 to 38% have been observed in OIS (Morrison and Vila, 1998; Morrison and Garner, 2011; Donner and Popovich, 2018), which is in stark contrast to the almost 90% hit rate reported in range-based annual firearms qualifications (Anderson and Plecas, 2000; Brown et al., 2021). In OIS incidents, officers have also reported experiencing perceptual distortions, impaired cognitive function, and reduced motor dexterity (e.g., Honig and Sultan, 2004; Artwohl, 2008; Klinger and Brunson, 2009). For example, Artwohl (2008) surveyed 157 police officers within a few weeks of being involved in an OIS. Findings demonstrated that most officers experienced perceptual narrowing, including diminished sound (84%) and tunnel vision (79%), and that the majority of officers (74%) responded on automatic pilot (i.e., with little or no conscious thought). Other studies have reported similar findings (e.g., Honig and Sultan, 2004; Klinger and Brunson, 2009).

Attentional control theory adds additional explanatory power to understanding performance impairments (Eysenck et al., 2007). This theory suggests that when exposed to a threatening stimulus, attention is drawn (or distracted) away from task relevant processes (e.g., decision-making) to the threat-related stimuli *via* psychological and neurophysiological responses (Eysenck et al., 2007; Nieuwenhuys and Oudejans, 2017; Di Nota and Huhta, 2019). Since attentional capacity is limited, it is difficult to attend to two things at the same time (Vickers, 2007). Therefore, when attention is focused on the threat, cognitive overload is more likely to occur, resulting in less attention available for mental and perceptual-motor processing (Driskell and Johnston, 1998; Eysenck et al., 2007; Hope, 2016).

These attentional, perceptual, and stress reactivity-related deficits mean that when presented with a threat, officers may be more prone to compromised performance, decision-making errors, and perceptual challenges (e.g., missing relevant cues, such as a subject pulling out a cellphone, not a gun; Easterbrook, 1959; Driskell and Salas, 1996; Vickers, 2007). Overall, the effects of stress on performance may be particularly detrimental during a critical incident, when officers are expected to demonstrate sound judgment and proficient performance. However, research in this area is limited, as studies tend to examine only narrow aspects of performance (e.g., shooting accuracy), use subjective measures of stress (e.g., self-report), or lack robust methods and measures for assessing performance.

### Impact of Training and Experience on Stress Reactivity

While the body’s default response to successfully deal with a threat is to stimulate the fight-or-flight response (LeDoux and Pine, 2016), training and experience are thought to moderate stress by intervening immediately following the initial autonomic stress response (Driskell and Salas, 1996; Kavanagh, 2005; Wollert et al., 2011). Theoretically, training and experience improve one’s ability to cope with a threat, subsequently affecting the appraisal process, which sustains and moderates the fight-or-flight physiology (Driskell and Salas, 1996; Anshel et al., 1997; Kelley et al., 2019).

Research provides mixed evidence for this theory (Rimmele et al., 2007; Johnson et al., 2014; Landman et al., 2016b). For example, during UoF simulation studies, officers on specialized and tactical teams displayed lower HR during a high-pressure scenario as compared to general duty officers (Landman et al., 2016b; James et al., 2020). In contrast, when Baldwin et al. (2019) examined officers’ level of operational skills training and years of experience, neither significantly modulated stress reactivity during general duty calls for service. Instead, stress reactivity was primarily associated with situational risk factors, such as the priority of the call and whether weapons were reported, or force was used. While the evidence is limited and mixed, greater levels of on-the-job experience and police training should, theoretically, improve coping and resilience to stressors, that is the very reason why training exists.

### Impact of Training and Experience on Performance Under Stress

Research demonstrates that a wide range of training techniques can improve performance, even under stressful conditions. For example, there are many training strategies that can enhance the acquisition, retention, and application of knowledge, skills, and abilities (KSAs), such as the use of spaced practice and providing appropriate feedback (Jenkins et al., 2021; Di Nota et al., 2021a; Bennell et al., 2021b). In addition, meta-analyses and systematic reviews across many domains (e.g., sport, military, medicine, policing) consistently identify the performance benefits produced by training under pressure or threat that replicates the operational environment (Kent et al., 2018; Gröpel and Mesagno, 2019; Low et al., 2021). As a result, contemporary operational police skills training now often includes scenario-based training (SBT) that gradually exposes officers to stress-inducing simulated encounters in an attempt to develop stress-resilient skills and performance (Reaves, 2016). Meta-analyses provide empirical support for this training approach as a way of improving performance (Saunders et al., 1996; Low et al., 2021). Accordingly, we expect that officers with higher levels of operational skills training will perform better than those with less training, as they will have had greater opportunity to acquire and practice their KSAs in SBT, making the KSAs more adaptive and stress resilient.

In addition to training, operational experience may also be important to performance and decision-making under stress. For example, through the acquisition and automation of schemas, which are forms of tacit knowledge gained on-the-job or during training, experienced individuals can discern subtleties in their environment that may be imperceptible to novices (Kavanagh, 2006; Kahneman and Klein, 2009; Klein, 2015). Using this tacit knowledge, the recognition-primed decision-making (RPDM) model suggests that under dynamic and complex circumstances, experienced decision-makers can quickly assess situations and draw on their schemas to evaluate options and determine the first workable solution through satisficing (Klein, 1997, 1999; Ward et al., 2011). RPDM is resilient to stress and more adaptable to complex and dynamic situations (Klein, 2015). Accordingly, studies have found that greater levels of policing experience are related to things like flexible rather than serial decision-making (Boulton and Cole, 2016), anticipation and cue recognition (Vickers and Lewinski, 2012; Renden et al., 2015; Suss and Ward, 2018), and reduced lethal force errors (Vickers and Lewinski, 2012; Landman et al., 2016b). However, many of these studies dichotomize experience into expert (e.g., tactical officers) and novice (e.g., cadets) categories, which may not account for the broad spectrum of training that officers receive, nor do they disentangle the distinct effects of on-the-job experience compared to training.

### Current Study

In the current study, active-duty police officers participated in a complex, dynamic, and realistic lethal force scenario to examine whether performance was affected by the officer’s level of operational skills training, years of police service, and stress reactivity. The findings will speak to the level of performance under stress that can reasonably be expected from officers, based on their current police training and experience. This will allow us to recommend evidence-based enhancements to training, as well as to inform the objective reasonableness standard used in courts of law.

More specifically, we hypothesized the following:

1. Officers will display elevated stress reactivity in response to the scenario, commensurate with those observed in naturalistic UoF encounters. Elevated stress reactivity is operationalized as an increase in sympathetic activity and a withdrawal of parasympathetic activity, measured by HR and HRV, as well as an increase in self-reported perceptual and cognitive distortions;
2. Stress reactivity will vary as a function of level of police training and years of police service;
3. Highly elevated stress reactivity will be associated with poorer performance, as operationalized by performance scales and lethal force errors; and
4. Higher levels of training and experience will be associated with better performance.

## Materials and Methods

### Participants

In June 2018, 122 active-duty police officers from a large Canadian police agency volunteered to participate in our study. The inclusion criteria for participants were that they were considered “fit for duty”^1^ by their police agency and currently on active duty. Table 1 shows the basic sociodemographic characteristics of the sample (*N* = 122).

**Table 1:** Participant demographics.

	*n*	%	*M*	*SD*
**Gender**				
Female	23	18.9		
Male	99	81.1		
**Age**			38.2	8.2
**Highest level of education completed**				
High school diploma or equivalent	10	8.2		
Apprenticeship/Trade school	5	4.1		
Some college	16	13.1		
College diploma or certificate	28	23.0		
Some university	17	13.9		
Bachelor’s degree	38	31.1		
Post-graduate certificate	2	1.6		
Master’s degree	5	4.1		
Doctoral degree	1	0.8		
**Current police rank**				
Reserve constable	1	0.8		
Constable	85	69.7		
Corporal	24	19.7		
Sergeant	10	8.2		
Staff sergeant	1	0.8		
Inspector	1	0.8		
**Years of police service**			11.2	6.6
**Previous experience with other police agency or the military**				
Yes	16	13.1		
No	106	86.9		
**Have you ever been involved in a lethal force encounter**				
Subject officer	5	4.1		
Witness officer (i.e., officer on scene)	8	6.6		
No	109	89.3		

### Materials

#### Demographic Questionnaire

A demographic questionnaire was used to collect age, gender, years of service, law enforcement experience, training, self-reported cardiovascular disease, and whether they were taking medication that could affect the cardiovascular system. Frequency of alcohol, tobacco, and caffeine consumption, as well as frequency of exercise, was also collected.

#### Stress Reactivity Monitoring Devices

Stress reactivity was measured using two Polar V800 Heart Rate Monitor Watches® and a Polar H7 Chest Strap Heart Rate Monitor® (Polar Electro Oy, Kempele, Finland). Together, these devices continuously record HR and R-R intervals (i.e., beat-to-beat intervals), with a sampling rate of 1,000 Hz for HRV analysis. These devices have been used in prior research when officers are on-shift or participating in realistic scenarios (Hope et al., 2016; Landman et al., 2016a; Baldwin et al., 2019). They have also been validated against hospital-grade electrocardiograms (ECG; Caminal et al., 2018; Gilgen-Ammann et al., 2019; Cilhoroz et al., 2020; Hernández-Vicente et al., 2021).^2^

#### Firearms Training System

Participants were equipped with a StressVest®, which is a non-projectile system that facilitates realistic scenario-based firearms training.^3^ Participants wear the StressVest® and a StressX® PRO Belt. Duty pistols are converted to fire a laser pulse that activates the StressVest® when it strikes center mass, the side, or head (with additional side panels and face sensor baseball hat). When hit, the StressX® PRO Belt delivers either a vibration or shock to the abdomen of the participant. The system has been shown to elicit stress reactivity, as measured by HR, commensurate to training with non-lethal training ammunition (i.e., Simunition® FX marking cartridges; Condon, 2015).

#### Video Recording Devices

In order to code participant performance, each scenario was video recorded by three ©GoPro HERO4 Silver cameras affixed in central locations around the study area. All participants also wore an eye tracker (©Applied Science Laboratories Mobile Eye-5 Glasses) and certain participants wore body worn cameras (Axon Body2®) for purposes unrelated to the current study. The video footage was used to provide multiple angles to assess performance throughout the scenario.

### Measures

#### Phase of the Scenario

As described in more detail in the Supplementary Material A (https://osf.io/qj2cg/), participants were exposed to a lethal force scenario. The scenario occurred in a building that had been designed to appear as an apartment complex in a rural setting. All participants were dispatched to a second-floor apartment for a call from a female complainant indicating that a male subject had been drinking heavily and was in breach of his probation conditions. At that point, the facilitator said, “scenario on” and participants had the opportunity to ask dispatch for additional information, if they chose to do so.

Upon arriving “on scene” and knocking on the door of the residence, the participant was greeted by a bystander, who indicated that the subject had committed an assault. The bystander remained in the scenario room and demanded the participant remove the subject, who was seated at the dining room table at the other end of the room. A partially obscured knife was on the table and the subject eventually drew it and put it to his throat, threatening to die by suicide. After some time passed, regardless of how much the officer attempted to verbally de-escalate or intervene, the subject ultimately complied and threw the knife on the ground towards the participant.

The scenario was allowed to naturally unfold a little longer until the subject spontaneously pulled a firearm, stood up, and started to shoot at the participant. This resulted in a lethal force response from the participant. Once shot at by the participant, the subject feigned a gunshot wound to the chest while the bystander contemporaneously produced and pointed a cellphone, verbally indicating that they were video recording the situation. Participants were then provided the opportunity to prioritize and perform whatever actions they deemed necessary (e.g., request resources, secure weapons, physically restrain subject and/or bystander, search subject, administer first aid). The scenario was allowed to come to a natural conclusion and was ended by the facilitator when the participant failed to demonstrate any new actions or strategies.

For the purpose of analyzing cardiovascular stress reactivity, the scenario (*M* = 9:25 min; *SD* = 2:32) was broken down temporally into five phases: (1) dispatch phase – from beginning of the simulated dispatch call to the facilitator saying “scenario on” (*M* = 1:10 min; *SD* = 0:53), (2) approach phase – from the facilitator saying “scenario on” to the bystander opening the apartment door (*M* = 0:46 min; *SD* = 0:26), (3) encounter phase – from the bystander opening the apartment door to the participant recognizing the knife on the table and/or the subject grabbing the knife on the table (*M* = 1:44 min; *SD* = 1:22), (4) critical phase – from the participant recognizing the knife on the table and/or the subject grabbing the knife on the table to the participant making physical contact with the subject (e.g., arrest; *M* = 3:08 min; *SD* = 1:40), and (5) scene management and aftercare (SM&A) phase – from the participant making physical contact with the subject to the facilitator saying “scenario over” (*M* = 2:38 min; *SD* = 1:04).

#### Stress Reactivity

##### Cardiovascular Stress Reactivity

Empirical research supports the use of HRV as a noninvasive measure of psychological and physiological arousal (Berntson and Cacioppo, 2004; Appelhans and Luecken, 2006; Thayer et al., 2012). Thus, HR and HRV were captured using monitoring devices. Data were entered into ©Kubios HRV Premium Version 3.3.1. (Biomedical Signal Analysis Group, Department of Applied Physics, University of Kuopio, Finland), which is research software for the analysis of HRV. Samples were created for each phase of the scenario.

The PNS Index and SNS Index, computed in ©Kubios HRV software, were used as a measure of stress reactivity in this study (Sahoo et al., 2019). These indices have been used in other research (James et al., 2020; Giuseppe et al., 2021; Lundell et al., 2021). See Supplementary Material B (https://osf.io/hf9p6/) for further details on the measures and methods used.

##### Perceptual and Cognitive Distortions

To examine whether the scenario resulted in perceptual and cognitive distortions – an indicator of stress reactivity – a 14-item questionnaire adapted from Artwohl (2008) was administered. Each perceptual and cognitive distortion during-scenario (10 items) and post-scenario (four items) was rated on a four-point Likert-type scale ranging from 0 “not at all” to 3 “to a great extent.” Total scores could range from 0 to 42. Perceptual and cognitive distortion scores were expressed as a percentage of the total possible score (42). See Supplementary Material C (https://osf.io/nawhm/) for a list of perceptual and cognitive distortions and descriptions.

#### Training

Participants’ training records and the training information captured in the demographics form were used to identify and assess their level of in-service operational skills training. Eight levels of training, from basic to elite, were established based on recency, frequency, and type of training experience participants received (see Table 2). See Supplementary Material D (https://osf.io/4f8er/) for details on the agency’s training and methods for categorizing the level of training.

**Table 2:** Level of training.

Order	Training level	Amount/type of training	*n*	%
8	Elite (level 2)	Emergency response team (i.e., tactical team)	14	11.5
7	Elite (level 1)	Use of force instructor	16	13.1
6	Advanced	Specialized (i.e., air marshal, crisis negotiator) or firearm instructor	12	9.8
5	Intermediate (level 3)	>5 Courses	10	8.2
4	Intermediate (level 2)	5 Courses	25	20.5
3	Intermediate (level 1)	4 Courses	20	16.4
2	Novice/basic (level 2)	3 Courses	17	13.9
1	Novice/basic (level 1)	2 Courses	8	6.6

#### Performance Metrics

To provide a robust assessment of performance, a combination of objective and subjective measures (Di Nota et al., 2021c) from four separate performance metrics were used: (1) the Deadly Force Judgment and Decision-Making (DFJDM), Tactical Social Interaction (TSI), and Crisis Intervention Team (CIT) metrics (Vila et al., 2018), (2) the agency’s performance metric, (3) the Scenario Training Assessment and Review (STAR) scale (Wollert et al., 2011), and (4) lethal force errors.

##### Deadly Force Judgment and Decision-Making, Tactical Social Interaction, and Crisis Intervention Team Metric

The DFJDM metric was developed to assess performance in situations requiring the UoF, whereas the TSI and CIT metrics were developed for measuring performance during police–public interactions and encounters with people suffering from mental illness or who are in crisis, respectively. The DFJDM includes 105 performance indicators weighted from −6 (extremely negative impact on performance) to +6 (extremely positive impact on performance). The TSI has 78 performance indicators weighted from 1 (no impact on performance) to 7 (extremely positive impact on performance) and the CIT is comprised of 112 performance indicators ranging from −4 (strong negative impact on performance) to +4 (strong positive impact on performance).

In accordance with Vila et al.’s (2018) recommendations, the authors and a group of police trainers selected performance indicators from these three metrics that were applicable to the study scenario. This resulted in a total of 39 performance indicators from the DFJDM (20), CIT (14), and TSI (5) that were then combined into a single metric (see James et al., 2019). When rating performance, indicators were assessed as to whether they were applicable (1 – Yes; 0 – No) for each officer in the scenario. If applicable, each indicator was rated as achieved or not (1 – Yes; 0 – No; Vila et al., 2018). Weighted performance scores were then expressed as a percentage of the potential weighted score for each officer in the scenario. Where a performance indicator was not applicable, it was removed from the potential score to avoid penalizing an officer for something they could not have done (e.g., assessing ability to reload firearm, when a reload was not necessary). See Supplementary Material E (https://osf.io/4gzyd/) for list of performance indicators and weightings.

##### Agency Metric

All items contained within the agencies’ performance rubrics for scenario-based training and the basic trauma equipment course were adapted into a single metric. This contained 44 items, including professionalism, law, and policy (three items), skills and techniques (five items), tactics and officer safety (28 items), and medical response (eight items). Each item was equally weighted and scored as (1 – Yes; 0 – No; Not applicable). Performance scores were expressed as a percentage of the potential applicable scores for each officer in the scenario. See Supplementary Material F (https://osf.io/8mfpq/) for a list of performance indicators.

##### Scenario Training Assessment and Review Scale

The Federal Law Enforcement Training Centre (FLETC) developed the STAR scale. The scale identifies eight factors considered essential to an officer’s operational performance, including: (1) situational awareness, (2) threat identification, (3) initial response, (4) scene control after the initial response, (5) application of force, (6) arrest/processing techniques, (7) communication, and (8) articulation/after action review (Wollert et al., 2011). Each item is rated on a four-point scale (1 – Not acceptable; 2 – Least desirable; 3 – Acceptable; 4 – Desirable; Not applicable). Performance scores were expressed as a percentage of the potential applicable scores for each officer in the scenario. See Supplementary Material G (https://osf.io/gmpx3/) for a list of performance indicators, ratings, descriptions, and modifications.

##### Lethal Force Errors

To evaluate lethal force errors, participants were assessed for whether they: (1) shot the subject while they were armed with a knife and exhibiting a threat of self-harm (i.e., decision-making error), or (2) shot the bystander who quickly produced and pointed a cellphone after the subject was shot, while verbally indicating that they were video recording the situation (i.e., mistake of fact error).

##### Overall Performance

To develop an overall performance measure, the average of the (1) DFJDM, TSI, and CIT metric, (2) agency metric, and (3) STAR scale was calculated.

##### Performance Coding and Reliability

A team of eight UoF subject matter experts and trainers coded participant performance using the metrics described above. All coders had received the agencies’ 3-week UoF instructor course and had extensive UoF training and/or review experience. Coders received 4 h of initial training on the use of the metrics and then completed four training assessments to confirm consistency and clarify metrics, where necessary. Coders were then randomly assigned to pairs and assigned a quarter of participants at random. Using scenario video footage, performance metrics for every participant were independently assessed by two coders to allow inter-rater reliability to be assessed.

Intraclass correlation coefficient (ICC) estimates for the total scores on each of the performance scales and their 95% CI were calculated. The resulting ICCs from the DFJDM, TSI, and CIT metric (ICC = 0.75, 95% CI [0.65–0.83]) and agency metric (ICC = 0.74, 95% CI [0.63–0.82]) were in the good–excellent (Cicchetti, 1994) or moderate–good range (Koo and Li, 2016). This indicated that coders had a relatively high degree of agreement and suggests that performance was rated similarly across coders. The STAR scale demonstrated poor-good (Cicchetti, 1994) or poor-moderate agreement (Koo and Li, 2016; ICC = 0.52, 95% CI [0.32–0.66]). To resolve discrepancies and achieve a single “most correct” assessment, independent third-party resolution was completed by another member of the coding team – neither of the original two coders (Syed and Nelson, 2015; Bakeman and Goodman, 2020). Once the independent third-party resolution was completed, the overall performance measure was calculated.

### Procedure

Before beginning the study, participants reviewed and signed an informed consent form. They were then equipped with cardiovascular monitoring devices and completed the demographics questionnaire. Next, participants were outfitted with other relevant equipment, including a StressVest™ system, eye-tracker, BWC, and all the inert tools they carry in the field. They were then exposed to the lowest shock level from the StressVest™. The shock was then increased to the highest “extreme” level, which the participants were informed they would experience, if shot, during the scenario. They then completed the scenario, which was facilitated by an expert police trainer, who remained with them throughout the entirety of the scenario to act as radio dispatch and ensure their safety and that of the role players. See Supplementary Material A (https://osf.io/qj2cg) for a detailed design and description of the scenario.

After the scenario, participants were de-equipped and completed the self-reported perceptual and cognitive distortions questionnaire. A random subsample of participants were recruited to wear a HR monitor to establish a true resting heart rate during sleep. Subsequently, participants were then debriefed by the researchers and a facilitator. After the debriefing, participants were compensated with a $50.00 gift card and those who volunteered to wear the heart rate monitor while sleeping, were compensated with an additional $50.00 gift card. All participants were provided the opportunity to withdraw their data, but none chose to do so.

The study was approved by the Carleton University Ethics Committee for Psychological Research (CUREB-B Clearance # 108733), as well as the Research Review Board (2018-04) of the agency from which the officers were recruited.

### Data Analyses

All measures for the current study were entered into SPSS v.27 (IBM Corp, 2020) for quantitative analysis. All dependent variables were examined for expected ranges and the presence of extreme outliers. The normal distribution of dependent variables was tested using the Kolmogorov–Smirnov test, as well as an examination of histograms and Q-Q plots. All performance scales, self-reported perceptual and cognitive distortions, and heart rate measures were normally distributed. SNS and PNS indexes had nonparametric distributions.

Paired-samples *t* tests were used to test the mean difference between paired observations. Independent-samples *t* tests and Mann–Whitney U tests were used to compare parametric and nonparametric measures between independent samples, respectively. Correlations between variables were assessed using Pearson’s correlation (*r*) for parametric distributions or Spearman’s rank correlation (*r_s_*) for nonparametric distributions.

For repeated measures with normal distributions, General Linear Model repeated measures ANOVA were used. Greenhouse–Geisser corrected value of *p* were reported when the assumption of sphericity was violated, as indicated by the Mauchly test. Significant main effects were further analyzed with Bonferroni-corrected *post hoc* tests. For nonparametric repeated measures, the Friedman test was used. Significant main effects from the Friedman test were further analyzed with Bonferroni-corrected Wilcoxon signed-rank tests and effect sizes calculated in accordance with Pallant (2010, p. 232). To examine the effect of training on HR, self-reported perceptual and cognitive distortions, and performance, one-way between-subjects ANOVAs were conducted. Kruskal–Wallis one-way ANOVAs were conducted to examine nonparametric HRV measures.

Multiple regression analysis was used to determine the relationships among stress reactivity, experience, and training on performance. To examine the two lethal force errors with dichotomous outcomes, logistic regression was used to model the data. All assumptions were met for regression analyses.

## Results

### Stress Reactivity in Response to the Scenario

To measure elevated stress reactivity, we first established a true resting heart rate with a subsample (*n* = 29) who wore a HR monitor to sleep. A paired-samples *t* test was conducted to compare HR_rest_ at the lowest 1 min while completing paperwork pre-scenario to HR while the officer was sleeping. As expected, HR_rest_ (*M* = 77.11, *SD* = 10.76) was significantly higher than HR while the officer was sleeping [*M* = 55.80, *SD* = 6.53, *t*(28) = 13.665, *p* < 0.001, *d* = 2.54]. HRrest for the full sample was 75.17 bpm (*SD* = 11.13; see Table 3), which is in line with the resting rate found for officers (pre-scenario) in similar studies (Andersen et al., 2018), although it is 10–15 bpm higher than on-duty HRrest (Anderson and Plecas, 2000; Baldwin et al., 2019). The slight elevation may be attributed to factors such as anticipatory stress while waiting for the scenario or the officer’s body positioning during the recording (e.g., sitting upright in a chair; Miles-Chan et al., 2013).

**Table 3:** Cardiovascular stress reactivity during sleep, while at rest, and during the phases of the scenario.

	HR_mean_ (bpm)			HR_max_ (bpm)			SNS index			PNS index		
	*M*	*SD*	*n*	*M*	*SD*	*n*	*M*	*SD*	*n*	*M*	*SD*	*n*
Sleep	55.80	6.17	29	-	-	-	−0.76	1.26	29	1.43	1.59	29
Resting	75.17	11.13	122	-	-	-	1.46	1.67	122	−0.78	1.23	122
Phase of scenario												
Dispatch	103.95	16.39	122	115.94	16.68	122	5.05	2.94	119	−2.46	0.81	119
Approach	124.11	16.56	122	139.76	16.13	122	9.76	4.53	119	−3.30	0.67	119
Encounter	130.32	20.04	122	142.16	17.43	122	10.13	5.46	119	−3.40	0.79	119
Critical	132.38	19.57	121	149.81	18.03	122	9.55	4.68	116	−3.51	0.70	116
SM&A	128.93	18.28	117	143.52	18.47	117	9.16	4.64	118	−3.35	0.80	118
Overall Scenario	128.98	18.11	122	152.50	17.23	122	7.80	3.39	116	−3.39	0.69	116

bpm, beats per minute and SM&A, scene management and aftercare.

Table 3 presents cardiovascular stress reactivity data for officers across the scenario. In support of the first hypothesis, the results indicate that participants experienced elevated stress reactivity during the scenario. HR_mean_scenario_ was 129 bpm (*SD* = 18.11). Elevated SNS Index_scenario_ (*M* = 7.8, *SD* = 3.39) and decreased PNS Index_scenario_ (*M* = −3.39, *SD* = 0.69) were also observed throughout the scenario. Average participant HR_max_critical_ was 149.81 (*SD* = 18.03), consistent with the HR reported during real world UoF encounters (Baldwin et al., 2019).

**Figure 1:**
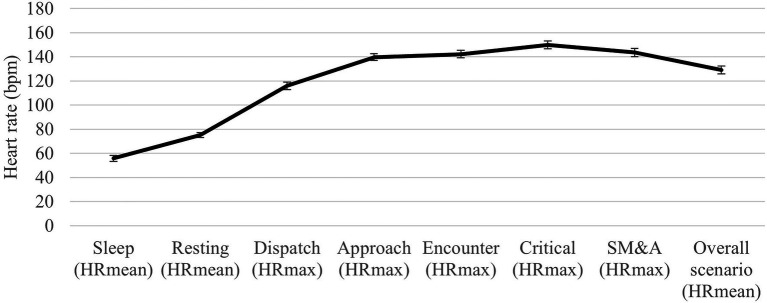
Heart rate (HR) during sleep, while at rest, and during the phases of the scenario. bpm, beats per minute and SM&A, scene management and aftercare. 95% CI error bars displayed.

Further supporting the first hypothesis, a repeated measures analysis (*n* = 117) demonstrated significant differences from at-rest HR and HR across the phases of the scenario [*F*(3.251, 377.126) = 1091.954, *p* < 0.001, *η_p_*^2^ = 0.90]. After a Bonferroni *post hoc* correction (*α* = 0.05/5 = 0.01) was applied, it revealed that HR_max_critical_ (*M* = 150.12, *SE* = 1.67) was significantly higher than HR_rest_ (*M* = 74.89, *SE* = 1.02, *p* < 0.001, *d* = 4.64), as well as HR_max_dispatch_ (*M* = 115.99, *SE* = 1.57, *p* < 0.001, *d* = 2.41), HR_max_approach_ (*M* = 139.87, *SE* = 1.51, *p* < 0.001, *d* = 0.96), HR_max_encounter_ (*M* = 142.41, *SE* = 1.67, *p* < 0.001, *d* = 0.80), and HR_max_SM&A_ (*M* = 143.52, *SE* = 1.71, *p* < 0.001, *d* = 0.59). See Figure 1 for a line chart of HR during sleep, while at rest, and during the phases of the scenario.

**Figure 2:**
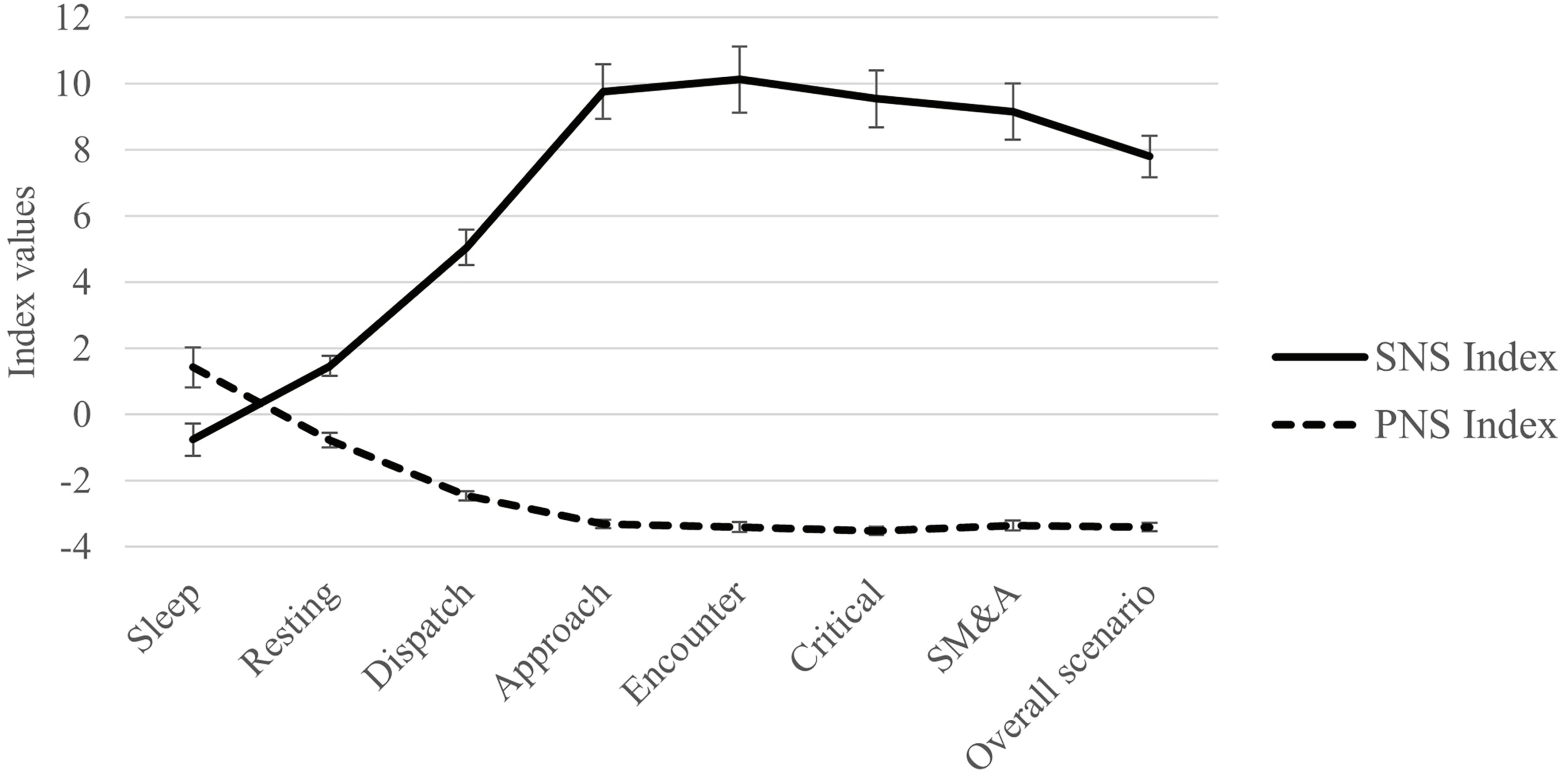
Sympathetic nervous system (SNS) and parasympathetic nervous system (PNS) index values during sleep, while at rest, and during the phases of the scenario. SM&A, scene management and aftercare. 95% CI error bars displayed.

Similarly, there was a statistically significant difference in SNS and PNS index values (*n* = 111) while at rest and across the phases of the scenario, *χ*^2^(5) = 316.86, *p* < 0.001 and *χ*^2^(5) = 370.77, *p* < 0.001, respectively. A *post hoc* analysis with Wilcoxon signed-rank tests was conducted with a Bonferroni correction (*α* = 0.05/5 = 0.01). There were large significant increases in SNS Index_critical_ compared to SNS Index_rest_ (*z* = 9.347, *p* < 0.001, *r* = 0.61) and SNS Index_dispatch_ (*z* = 8.294, *p* < 0.001, *r* = 0.54). However, there were no statistically significant differences between SNS Index_critical_ and SNS Index_approach_, SNS Index_encounter_, or SNS Index_SM&A_ (*p* > 0.01, *r* = ±0.04–0.09). There were small to large significant decreases in PNS Index_critical_ compared to PNS Index_rest_ (*z* = −9.347, *p* < 0.001, *r* = −0.61), PNS Index_dispatch_ (*z* = −9.199, *p* < 0.001, *r* = −0.61), PNS Index_approach_ (*z* = −9.199, *p* < 0.001, *r* = −0.27), and PNS Index_SM&A_ (*z* = −2.855, *p* = 0.004, *r* = −0.19). However, once Bonferroni-corrected, there was a small nonsignificant difference between PNS Index_critical_ and PNS Index_encounter_ (*z* = −2.334, *p* = 0.02, *r* = −0.15). Overall, these results provide support for our first hypothesis. See Figure 2 for a line chart of SNS and PNS index values during sleep, while at rest, and during the phases of the scenario.

To further assess the first hypothesis, we examined perceptual and cognitive distortions experienced by participants. The majority of participants reported experiencing the sensation of being on automatic pilot (90.9%), tunnel vision (87.6%), heightened visual clarity (82.6%), and diminished sound (70.2%) during the scenario (see Table 4). Overall, the mean perceptual and cognitive distortion score for participants was 33.6% (*SD* = 15.9), indicating a notable presence of distortions. These results also provide support for our first hypothesis. Perceptual and cognitive distortion scores were not significantly associated with cardiovascular stress reactivity (i.e., HR, SNS, and PNS index values) during the overall scenario and during the critical phase of the scenario (*p* > 0.05).

**Table 4:** Self-reported perceptual and cognitive distortions experienced by participants during- and post-scenario.

	Not at all		Very little		Somewhat		To a great extent	
	*n*	%	*n*	%	*n*	%	*n*	%
Diminished sound (i.e., inability to hear very loud sounds you would ordinarily hear, such as gunshots)	36	29.8	44	36.4	35	28.9	6	5.0
Intensified sounds	41	33.9	45	37.2	30	24.8	5	4.1
Heightened visual clarity	21	17.4	44	36.4	49	40.5	7	5.8
Tunnel vision (i.e., loss or narrowing of peripheral vision)	15	12.4	30	24.8	55	45.5	21	17.4
Automatic pilot (i.e., I responded with little or no conscious thought)	11	9.1	34	28.1	53	43.8	23	19.0
Slow motion time (i.e., time slowed down)	52	43.0	41	33.9	24	19.8	4	3.3
Fast motion time (i.e., time sped up)	49	40.5	30	24.8	29	24.0	13	10.7
Temporary paralysis (i.e., froze)	65	54.2	32	26.7	21	17.5	2	1.7
Dissociation (i.e., a sense of detachment or unreality)	72	60.0	22	18.3	25	20.8	1	0.8
Intrusive distracting thoughts	106	87.6	6	5.0	9	7.4	0	0.0
Memory loss for part of the event	36	30.0	49	40.8	33	27.5	2	1.7
Memory loss for some of my own behavior	33	27.3	51	42.1	35	28.9	2	1.7
Memory distortions	65	53.7	38	31.4	15	12.4	3	2.5
“Flashbulb” memories	41	33.9	29	24.0	37	30.6	14	11.6

#### Stress Reactivity as a Function of Training and Experience

**Figure 3:**
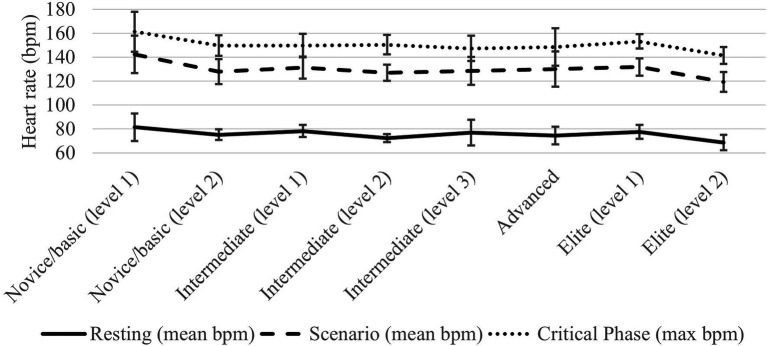
Heart rate as a function of level of training. bpm, beats per minute. 95% CI error bars displayed.

To test the second hypothesis, a one-way between-subjects ANOVA was conducted to examine the effect of training on stress reactivity. There were small nonsignificant effects of training on HR_rest_, HR_mean_scenario_, and HR_max_critical_. Nonsignificant results were also observed when conducting a Kruskal–Wallis one-way ANOVA for training on SNS Index_scenario_, PNS Index_scenario_, SNS Index_critical_, and PNS Index_critical_ (*p* > 0.05). These results failed to support our second hypothesis, as stress reactivity was similar across levels of training. See Figure 3 for baseline and scenario heart rate as a function of training.

To examine whether years of police service was associated with stress reactivity, a series of nonparametric correlation tests were conducted with HR, SNS, and PNS index values at rest, during the scenario, and during the critical phase of the scenario. Once Bonferroni-corrected (*α* = 0.05/7 = 0.007), years of service was significantly associated with HR_mean_scenario_ (*r_s_* = −0.26, *p* = 0.005), HR_max_critical_ (*r_s_* = −0.35, *p* < 0.001), and PNS Index_critical_ (*r_s_* = 0.26, *p* = 0.005).

A one-way between-subjects ANOVA revealed a small non-significant effect of training on perceptual and cognitive distortion scores [*F*(7, 113) = 1.585, *p* = 0.147, *η*^2^ = 0.089]. Years of police service was also not significantly associated with perceptual and cognitive distortion scores (*r_s_* = −0.10, *p* = 0.258). These mixed results regarding the effect of experience on stress reactivity provide some support for Hypothesis 2.

#### Performance as a Function of Stress Reactivity

All performance metrics had average scores that ranged from 50 to 66%, arguably indicating suboptimal performance under stress. Large positive correlations (*r* > 0.60, *p* < 0.001) between the three performance scales were observed. See Supplementary Material H (https://osf.io/73c4p/) for descriptive statistics and correlation matrix for performance metrics.

To examine our third hypothesis, a series of correlations between the performance metrics and HR, HRV, and self-reported perceptual and cognitive distortions were calculated (see Table 5). HR and perceptual and cognitive distortions were not significantly associated with performance metrics (*p* > 0.05). SNS Index_scenario_ and SNS Index_critical_ demonstrated a trend suggesting that, as participants’ sympathetic activity increased, their performance decreased. Specifically, small to moderate negative correlations were found between SNS Index_critical_ and all performance metrics. However, once a Bonferroni correction for multiple comparisons per dependent variable was applied (*α* = 0.05/7 = 0.007), only the correlation with the STAR scale and the overall performance rating remained significant (*p* < 0.001). PNS Index_scenario_ and PNS Index_critical_, while not statistically significant, demonstrated small positive correlations with all performance metrics, suggesting that parasympathetic withdrawal may be associated with a deterioration in performance. Using G*Power (Faul et al., 2007), a compromise power analysis indicated that the study sample size (*n* = 116) was considerably underpowered (16–40% power) to detect a significant effect size of that magnitude (i.e., *r* = 0.09–0.16).

**Table 5:** Correlations between stress reactivity and performance metrics.

Performance scales	Overall scenario			Critical phase			Perceptual and cognitive distortions
	HR_mean_	SNS index	PNS index	HR_max_	SNS index	PNS index	
DFJDM, TSI, and CIT	0.00	−0.10	0.02	0.05	−0.22^*^	0.09	0.034
Agency metric	−0.02	−0.09	0.05	−0.03	−0.22^*^	0.12	−0.065
STAR scale	−0.08	−0.21^*^	0.13	−0.01	−0.30^***^	0.16	−0.004
Overall rating	−0.04	−0.16	0.08	0.01	−0.29^***^	0.15	−0.006

Perceptual and cognitive distortions (*n* = 121), HR (*n* = 122), and HRV (*n* = 116). An independent samples *t* test was conducted to examine if there were differences in performance between participants with and without HRV data. No significant differences were found (*p* > 0.05).

* Indicates *p* < 0.05.

*** Indicates *p* < 0.001.

#### Performance as a Function of Training and Experience

**Figure 4:**
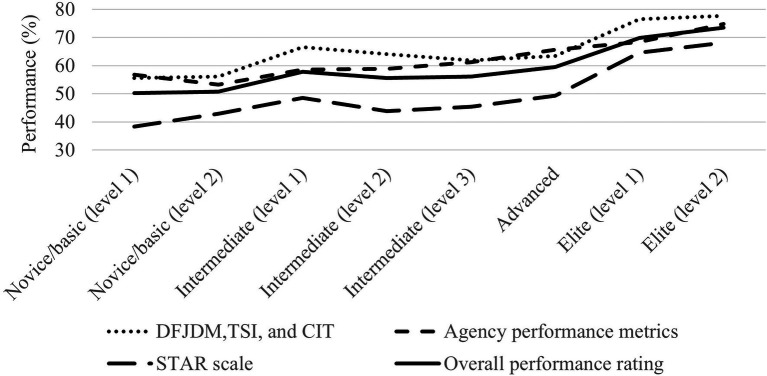
Performance metrics (%) as a function of level of training.

Our fourth hypothesis was tested with a one-way between-subjects ANOVA to compare the effects of the level of training on performance metrics. There was a significant moderate to large effect of training on DFJDM, TSI, and CIT [*F*(7, 114) = 3.495, *p* = 0.002, *η*^2^ = 0.177], agency performance metrics [*F*(7, 114) = 7.225, *p* < 0.001, *η*^2^ = 0.307], STAR scale [*F*(7, 114) = 5.928, *p* < 0.001, *η*^2^ = 0.267], and overall performance rating [*F*(7, 114) = 6.882, *p* < 0.001, *η*^2^ = 0.297]. Therefore, participants with higher levels of operational skills training displayed measurably better performance. See Figure 4 and Supplementary Material I (https://osf.io/zn6mr/) for performance scores across level of training. Years of police service was not significantly associated with any performance metrics (*r_s_* < 0.05, *p* > 0.05).

To examine the unique effects of training, experience, and stress reactivity on performance, multiple regression analysis was conducted. Due to high collinearity between cardiovascular measures (*r_s_* > ±0.80), and the nonsignificant correlations between performance and both HR and the PNS Index, only SNS Index_critical_ was retained in the model. In all four models (see Table 6), level of training had a significant effect on performance (*p* < 0.001), whereby for every increase in level of training (eight levels), there would be approximately a three unit (*Β* = 2.87–3.36) increase in each of the performance metrics (%). Conversely, for every increase in years of police service, performance metrics (%) decreased by approximately 0.39 (*Β* = 0.28–0.50), though this effect did not reach significance for either the DFJDM, TSI, and CIT (*p* = 0.093), or the agency performance metrics (*p* = 0.054). With regard to stress reactivity, for every one-unit increase in SNS Index_critical_ (*M* = 9.6, *SD* = 4.7), performance metrics (%) decreased by approximately 0.57 (*Β* = 0.22–0.99), though this effect did not reach a level of statistical significance for the DFJDM, TSI, and CIT (*p* = 0.145), or the agency performance metrics (*p* = 0.304).

**Table 6:** Multiple regressions for training, experience and stress reactivity on performance.

Predictors	Overall performance rating				DFJDM, TSI, and CIT				Agency performance metrics				STAR scale			
	*B*	SE B	*β*	*p*	*B*	SE B	*β*	*p*	*B*	SE B	*β*	*p*	*B*	SE B	*β*	*p*
Level of operational skills training	3.048	0.549	0.461	<0.001	2.914	0.744	0.353	<0.001	2.870	0.457	0.520	<0.001	3.359	0.742	0.386	<0.001
Years of police service	−0.393	0.175	−0.182	0.026	−0.401	0.237	−0.149	0.093	−0.282	0.145	−0.157	0.054	−0.496	0.236	−0.174	0.038
SNS Index_critical_	−0.571	0.255	−0.188	0.027	−0.507	0.345	−0.134	0.145	−0.219	0.212	−0.086	0.304	−0.986	0.344	−0.246	0.005
*R* ^2^	0.30				0.17				0.30				0.26			

Standardized coefficients indicated that the strength of the effect from the level of training (*b* = 0.35–52) on performance was approximately double that of years of police service (*b* = 0.15–18) and stress reactivity (*b* = 0.09–25). Overall, level of training, years of police service, and stress reactivity (SNS Index_critical_) explained approximately one quarter (*R*^2^ = 0.17–0.30) of the variance in performance in the scenario.

##### Lethal Force Errors

**Figure 5:**
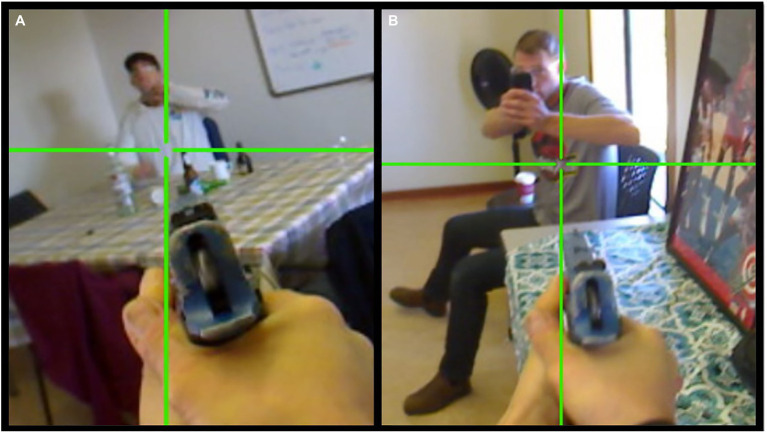
Lethal force errors during the scenario. **(A)** Shooting the subject while they were armed with a knife and exhibiting a threat of self-harm (i.e., decision-making error); and **(B)** shooting the bystander holding a cell phone (i.e., mistake of fact error). Green crosshairs represent participant gaze (from eye-tracker) at central mass while pulling the trigger.

A total of 34 (27.9%) participants made one or more lethal force errors during the scenario: nine (7.4%) shot the subject while they were armed with a knife and exhibiting a threat of self-harm (i.e., decision-making error); 20 (16.4%) shot the bystander who quickly produced and pointed a cellphone after the subject was shot, while verbally indicating that they were video recording the situation (i.e., mistake of fact error); and five (4.1%) made both errors (see Figure 5).

To examine whether training, experience, and stress reactivity predicted lethal force errors, logistic regression analysis was conducted (see Table 7). All independent variables predicted (*p* < 0.05) the subject being shot while they were armed with a knife and exhibiting a threat of self-harm. Specifically, for each increase in level of training, the odds of shooting the subject while they were armed with a knife and exhibiting a threat of self-harm increased by 37% and the odds increased 12% for every additional year of police service. An increase in stress reactivity (i.e., one-unit increase in SNS Index_critical_) also increased the odds of lethal force error on a subject armed with a knife and exhibiting a threat of self-harm by 25%. None of the variables significantly increased or decreased the odds of shooting the bystander who quickly produced and pointed a cellphone after the subject was shot. See Supplementary Material J (https://osf.io/2srpu/) for a breakdown of level of training by type of lethal force error.

**Table 7:** Logistic regressions for training, experience, and stress reactivity on lethal force errors.

Predictors	Subject^a^				Bystander^b^			
	Exp(B)	95% CI		Sig.	Exp(B)	95% CI		Sig.
		Lower	Upper			Lower	Upper	
Level of training	1.374	1.003	1.882	0.048	0.919	0.739	1.143	0.450
Years of police service	1.119	1.023	1.225	0.014	0.986	0.920	1.057	0.693
SNS index_critical_	1.247	1.069	1.454	0.005	1.008	0.907	1.120	0.888

a Shot the subject while they were armed with a knife and exhibiting a threat of self-harm (i.e., decision-making error).

b Shot the bystander who quickly produced and pointed a cellphone after the subject was shot, verbally indicating that they were video recording the situation (i.e., mistake of fact error).

### Exploratory Analysis of Behavioral Predictors of Performance

For an exploratory analysis of which individual behaviors were most associated with overall scores on the performance metrics, see Supplementary Material K (https://osf.io/vh2s6/).

## Discussion

Below, we briefly discuss the results related to each hypothesis and explore their implications.

### Stress Reactions to the Scenario

In support of Hypothesis 1, officers displayed significantly elevated stress reactivity in response to the scenario, including large increases in SNS arousal and PNS withdrawal, consistent with a threat response (Castaldo et al., 2015; Laborde et al., 2017). Self-reported perceptual and cognitive distortions and large increases in HR were also observed, commensurate with those reported in naturalistic UoF encounters (e.g., Anderson et al., 2002; Artwohl, 2008; Andersen et al., 2016). For example, officers’ cardiovascular stress reactivity during the critical phase of our scenario reached an average of 150 bpm (75 bpm higher than their pre-scenario resting rate). In comparison, Baldwin et al. (2019) reported stress reactivity in the range of 146 bpm when officers drew their firearm for the purpose of arresting a subject under threatening naturalistic conditions. Therefore, the HR produced during our scenario approximates with stress reactions to real world police encounters.

Over 70% of our participants also reported experiencing tunnel vision, heightened visual clarity, and diminished sound. These results closely correspond with the perceptual and cognitive distortions reported by others (Honig and Sultan, 2004; Artwohl, 2008; Klinger and Brunson, 2009). The high prevalence of perceptual distortions observed in this study also aligns with attentional control theory (Eysenck et al., 2007), which suggests that under stress, attention is directed toward the threatening stimuli, rather than task relevant processes (e.g., decision-making). These indications of perceptual narrowing are further supported by research showing that the perceptual field tends to shrink under stress (Vickers, 2007; Honig and Lewinski, 2008). Additionally, the majority of participants (91%) reported that they responded on automatic pilot. This corresponds with decision-making research which demonstrates that under dynamic and complex circumstances, responses rely heavily on intuition, which occur in an automatic manner (Kahneman and Klein, 2009; Ward et al., 2011; Klein, 2015). Our cardiovascular stress reactivity measures (i.e., HR and HRV) were not associated with self-reported perceptual and cognitive distortion scores, suggesting that self-reports of these distortions may not be a good proxy measure for stress reactivity, at least under high levels of stress. This further underscores the importance of collecting both objective and subjective measures of the phenomenon under study (Di Nota et al., 2021c).

Combined, the stress reactivity data indicate that the scenario developed for this study produced adverse physiological, attentional, and perceptual conditions. Thus, this scenario arguably provides reasonably realistic conditions under which to study and draw conclusions about what *might* happen to performance in highly stressful real-world police encounters. This is important, not only for assessing the efficacy of agency training, but also for informing the courts about how officers might reasonably perform when responding to a threat, given the current police training they have received. Further, the findings indicate how this performance and stress reactivity may be influenced by an officer’s level of current police training and experience.

### Impact of Training and Experience on Stress Reactivity

It is believed that training and experience can improve one’s ability to cope with a threatening stimuli, subsequently affecting the threat appraisal process, which sustains and moderates the fight-or-flight physiology (Driskell and Salas, 1996; Anshel et al., 1997; Kelley et al., 2019). The current study’s results provided mixed evidence for Hypothesis 2, which examined this relationship. Specifically, in contrast to what we expected, there was no effect regarding level of training on cardiovascular stress reactivity or the extent of perceptual and cognitive distortions experienced. Current findings correspond with Baldwin et al. (2019) who did not find an effect of level of training on physiological arousal when officers from the same agency as the current study responded to general duty calls for service. Together, these findings may indicate that the agency’s training does not include or sufficiently embed techniques that have been shown to promote adaptive coping mechanisms (e.g., mental rehearsal, reappraisal; Anshel, 2000; Colin et al., 2014). Another possible explanation is that the agency’s SBT is not currently eliciting significant enough stress reactivity to replicate the naturalistic environment and result in improved coping, advanced schemas, and stress resilient KSAs. While we are not proposing that all scenarios in SBT include high levels of stress, a progressive increase in stressful scenarios, once skills have been acquired, has shown benefits for performance that generalize across novel stressors and tasks (Driskell et al., 2001; Di Nota et al., 2021a).

Research also indicates that the threat response is malleable, with specific types of training being shown to increase one’s ability to control stress reactions (Arnetz et al., 2009; McCraty and Atkinson, 2012; Andersen et al., 2018). For example, using HRV biofeedback, Andersen and Gustafsberg (2016) and Andersen et al. (2016, 2018) taught officers to modulate autonomic arousal during threat inducing SBT by evoking parasympathetic activation. This autonomic modulation training resulted in lower maximum HR and quicker recovery from critical incident stress (i.e., the time it took to return to their average resting HR) following threat exposure (a measure of PNS activation; Thayer and Sternberg, 2006). Adopting autonomic modulation training, or embedding such techniques in already existing skills training, may better equip officers to adaptively modify their stress reactivity during real-world critical incidents, and ultimately improve performance under stress (Andersen et al., 2018; Bennell et al., 2021a).

In partial support of Hypothesis 2, we did find that more years of police service reduced parasympathetic withdrawal and HR in the critical phase and overall scenario, although no effect was observed for SNS arousal or perceptual and cognitive distortion scores. This provides some evidence that on-the-job experience may be important for parasympathetic regulation, which plays a role in forming a flexible response to environmental demands (Thayer et al., 2009; Roos et al., 2017; Andersen et al., 2018). It is unclear why this mixed effect with training and experience was observed, however, research has previously found that years of police experience influenced the extent to which officers believed they could cope with stressful events (Anshel et al., 1997). Further research exploring the role of training and experience is warranted.

### Impact of Stress on Performance

Given the large positive correlations that were observed between the three performance scales used, the average of the three scales was used to create an overall performance score, which captured a more comprehensive rating of KSAs essential to police work. Under the stressful conditions produced by our scenario, average participant scores for all performance scales ranged from 50 to 66%, arguably demonstrating suboptimal performance. However, it is important to note that due to the broad scope of performance indicators used in this study, it is likely beyond the ability of any officer to perform all expected tasks on their own. For example, many officers justifiably chose to prioritize providing medical care to the subject until back-up and emergency medical services (EMS) arrived. This would have resulted in lower scores for items related to scene management, such as securing weapons and evidence, which the officer may not have assessed as a priority given the circumstances (i.e., the subject suffering from a gunshot wound to the chest).

Additionally, under stress, over a quarter of officers made one or more lethal force errors during the scenario, including decision-making errors (7%), mistake of fact errors (16%), or a combination of the two (4%). Since our study did not have a control (i.e., low stress) scenario for comparison, we cannot determine the full extent to which these performance deficits and errors were stress induced. However, our study does show that SNS arousal during the critical phase of the scenario was associated with small to moderate decreases in performance, meaning that those who had higher, more maladaptive SNS arousal during the scenario displayed poorer performance than those with lower, more adaptive SNS arousal. Additionally, while it did not reach statistical significance, small effects were observed, suggesting that parasympathetic withdrawal may also be associated with a deterioration in performance. This trend adds to the growing evidence that indicates performance deficits may not only be related to maladaptive SNS arousal, but also the suppression of the stress modulating parasympathetic influence (Saus et al., 2006; Andersen et al., 2018; Spangler et al., 2018).

Even when controlling for level of training and experience, SNS arousal was still associated with performance deficits and increased odds of lethal force decision-making errors, though not mistake of fact error. With SNS Index values that ranged from 2 (low – more adaptive) to 25 (high – more maladaptive) during the critical phase of the scenario, model estimates indicate that maladaptive stress-induced deficits could decrease performance upwards of 5–23%, depending on the performance metric. Similarly, the odds of making a lethal force decision-making error would be 5.7 times higher for those with the highest SNS arousal, compared to those with the lowest SNS arousal. These findings and trends are consistent with real-world studies and scenario-based experiments, which demonstrate that maladaptive stress arousal can result in degradation of task accuracy, increased task errors, and deficits in motor skills and cognitive functions, such as perception, attention, and decision-making (e.g., Driskell and Salas, 1996; Johnston et al., 1997; Morrison and Vila, 1998). These findings provide strong evidence in support of Hypothesis 3.

Conversely, HR was not found to be associated with performance. Thus, while HR is the most easily monitored physiological proxy of stress, we must caution that this is not an absolute measure of an individual’s stress reactivity, nor does it unequivocally predict individual performance under stressful conditions, as HR is influenced by a variety of factors (Meyerhoff et al., 2004; Brisinda et al., 2015; Arble et al., 2019). Additionally, self-reported perceptual and cognitive distortion scores were not associated with performance, which may indicate that while they may be maladaptive for certain aspects of performance (e.g., situational awareness), they may also be adaptive for other aspects, such as officer safety. Therefore, caution should be used when inferring things about an individual’s in-the-moment performance based on post-incident self-reported distortions, particularly given what we know about memory distortions during stress and inaccuracies in self-reports (Di Nota et al., 2020). Based on these findings, future studies examining the relationship between stress and performance should use robust measures of stress reactivity (e.g., HRV, antithrombin), which have shown predictive value (e.g., Taverniers and De Boeck, 2014; Arble et al., 2019; James et al., 2020).

### Impact of Training and Experience on Performance Under Stress

In support of Hypothesis 4, there were moderate to large effects of training on all performance scales. For example, overall performance scores increased steadily from 50% for novice officers or those with basic training, to 74% for elite tactical officers. In fact, when controlling for years of police service and stress reactivity, training was the largest predictor of performance, with model estimates showing a 3% rise in performance for every increase in level of training (eight levels). This indicates that while overall performance was low, significant improvements in performance under stress can be achieved through greater levels of operational skills training.

Conversely, when controlling for training and stress reactivity, years of police service was negatively associated with performance, with model estimates showing that for every 5-year increase in years of service, performance decreased approximately 2%. This finding was somewhat unexpected as research shows that experience can improve performance, including decision-making and cue recognition (e.g., Renden et al., 2015; Boulton and Cole, 2016; Mangels et al., 2020). Since experience and training are inevitably related, our findings may be a result of using regression analysis to determine the distinct effect of on-the-job experience, while controlling for level of training. Our findings may then indicate that minimum qualifications and skills maintenance training, absent of additional or supplemental training and practice, are not sufficient to retain KSAs in the long-term (O’Neill et al., 2019). This may be particularly true for certain KSAs that are rarely used in the field, such as the UoF and medical care for a gunshot wound (Baldwin et al., 2020; Singh, 2020). Therefore, years of police service may be a crude measure of experience, as it is not necessarily indicative of exposure to critical incidents (Klein, 1999).

While greater levels of training improved global performance in the scenario, more advanced training, as well as higher years of police service, were both predictors of increased lethal force decision-making errors, even when controlling for stress reactivity. In contrast, with regard to the mistake of fact errors, neither training nor years of police service predicted shooting the bystander who quickly produced and pointed a cellphone after the subject was shot. These findings do not support Hypothesis 4, nor do they align with previous research that has shown a reduction of lethal force errors with greater levels of training and experience (Vickers and Lewinski, 2012; Landman et al., 2016b).

Research related to decision-making in naturalistic environments is helpful for understanding these unexpected results. According to this body of research, both the decision-making and mistake of fact lethal force errors observed in this study would be classified as rule-based (or misdiagnosis) errors (Reason, 2000; Taylor, 2019). This type of error involves an intended behavior (e.g., discharging a firearm at a perceived threat) that results in an unintended outcome (e.g., shooting an unarmed subject) due to a misdiagnosis of the situation and application of the wrong rule or schema (Taylor, 2019). Recall from our earlier description of RPDM that individuals rely heavily on cognitive shortcuts (e.g., satisficing) to quickly assess situations, evaluate options, and determine the first workable response (Klein, 1997, 1999; Kahneman and Klein, 2009). While this type of response is resilient to stress, requires less attentional resources, and enables a quick response to a perceived threat, it does not always result in the selection of the best response (Kahneman and Klein, 2009; Ward et al., 2011; Klein, 2015). Thus, with regard to the mistake of fact error, given the context of just being shot at by the subject, when the officers in our study saw the bystander quickly pulling an object from his pocket and raising it, this pattern was congruent with, and likely to be recognized as, a threat.

### Implications for Training

Given the sub-optimal performance observed in this study, it is recommended that LEAs and their trainers reflect on their current training and further incorporate evidence-based best practices from recent reviews (e.g., Jenkins et al., 2021; Di Nota et al., 2021a; Bennell et al., 2021b), in hopes of achieving better performance. Importantly, the exploratory analysis found in the supplemental material identified several behaviors that were highly associated with positive performance. These behaviors included things like assessing the situation, recognizing threat cues, competence with intervention options, de-escalation, and maintaining tactical advantage (i.e., time, distance, cover, concealment). Thus, greater integration and focus on these behaviors in training could result in positive impacts in overall performance.

Regarding the decision-making errors observed in this study, Andersen et al. (2018) cautioned against use of force models (and associated training) that may reinforce if-then contingencies, such as relating a weapon or the threat of grievous bodily harm or death to the use of lethal force. While it is certainly important for public and police safety for an officer to *draw* their firearm in response to a weapon or lethal threat in relevant instances, if use of force models do promote if-then thinking, maladaptive heuristics may be relied on that are inappropriate in certain circumstances. For instance, in our study, we observed a significant number of officers discharge their firearm at a subject who was armed with a knife but was exhibiting a threat of suicide. The odds of doing so also increased with more training and experience. Therefore, it is possible that the current UoF model and related training, are inadvertently creating and reinforcing inappropriate mental shortcuts that may be used under dynamic and highly stressful situations. Thus, LEAs should examine evidence-based training and models that target decision-making (e.g., Vickers, 2007; Klein and Borders, 2016; Engel et al., 2020) and problem-solving abilities (e.g., Rajakaruna et al., 2017; Belur et al., 2019; Blumberg et al., 2019).

Lastly, agencies need to ensure adequate amounts and frequency of training are provided to achieve mastery and retention of evidence-based KSAs (e.g., O’Neill et al., 2019; Bennell et al., 2021b; Di Nota et al., 2021b), as rehearsed and automated skills are influenced to a lesser degree by stress (Vickers and Lewinski, 2012; Renden et al., 2017; Arble et al., 2019). Training should also include appropriate amounts of representative practice that is commensurate with real-world settings, to allow officers the opportunity to practice and integrate a wide-range of KSAs under stressful conditions (e.g., tactics, de-escalation, decision-making, perceptual-motor movement, medical aftercare). Several studies have demonstrated that training under stress can improve police performance, enhance officer safety, and reduce use of force errors (e.g., Nieuwenhuys and Oudejans, 2011b; Taverniers and De Boeck, 2014; Andersen et al., 2018).

### Implications for the Objective Reasonableness Standard

Critics have argued that the objective reasonableness standard lacks an evidence-based foundation and focuses too much on the general dangers and stressful nature of policing (Fagan and Campbell, 2020; Zamoff, 2020). As the courts’ interpretation of what is reasonable is not static, research of the type reported here can advance the standard by “injecting a consistent dose of evidentiary rigor” (Zamoff, 2020, p. 585).

Performance under high levels of stress in this study was sub-optimal, with overall performance scores of 59% and over a quarter of officers making one or more lethal force errors during the scenario. While proper training may significantly improve performance, threat-induced performance deficits and lethal force errors in police officers are persistent, even with training (Nieuwenhuys et al., 2015). For example, even the sample of highly trained tactical officers in this study had performance scores of 74, and 14% made lethal force errors under stressful conditions, despite a quarter of their shift time being devoted to training (Cyr et al., 2020). These findings suggest that a reasonable officer, regardless of the amount of training and experience they have received, will likely not perform flawlessly under the unpredictable, novel, and potentially uncontrollable circumstances of a critical incident.

While this information is necessary to inform judgments concerning the reasonableness of an officer’s actions, the purpose of this research is not to excuse sub-optimal performance or errors by the police. Instead, the aim is to paint a realistic picture of human performance under stress, identify the extent to which current police training and experience can improve performance, and promote police accountability. Accordingly, the results suggest that unless there is a significant investment in more frequent and evidence-based training, police officers are likely not sufficiently prepared to deliver optimal performance in critical incidents, which can impact both public and police safety. Thus, absent of evidence of bias, malice, or gross incompetence on the part of an officer, responsibility for poor performance or lethal force errors lies with LEAs and governments who are responsible for setting evidence-based training standards and ensuring that they can be met. Currently, many police services identify significant barriers to providing training, such as limited funding, resources, and facilities (Rojek et al., 2020). At this critical juncture in time, when trust and confidence in policing are being significantly tested (e.g., Leger, 2021), a concerted effort is required to address these challenges.

### Study Limitations

While we are optimistic that our research findings can improve police training and inform the courts understanding of reasonable performance under stress, we caution readers to interpret and use the findings with consideration to various study limitations presented.

While the results of this study paint a stark picture of performance under stress, which may cause some alarm, these results must be considered within the context of what is *actually* occurring in the agency’s operational environment. For example, with over 16,000 officers policing approximately 8 million people, the agency’s OIS are relatively rare, with an average of 21 per year; accounting for 0.0008% of their police occurrences or one OIS in approximately 130,000 occurrences. These incidents also make up less than 1% of the number of times officers from the participating agency displayed or pointed their firearm at a subject, demonstrating that the vast majority of these high-risk situations are resolved without lethal force. Thus, while we can draw conclusions about what *might* happen to performance in highly stressful real-world police encounters, we must caution that it does not necessarily mean that it is occurring in naturalistic settings.

This study also involved only a single scenario, which was specifically designed to be complex and dynamic, and left the officer to respond on their own without backup. Such scenarios are known to elicit significant cognitive load (Mugford et al., 2013; Hope, 2016), which could inflate the sort of performance deficits we observed. While the scenario was designed to be as realistic as possible to cause high levels of stress in participants, it is also important to note that even a realistic scenario does not completely mirror the stress induced by a critical incident. For instance, in a training or research scenario, officers are aware they will not be seriously injured or killed, nor be subjected to post-OIS stressors (e.g., external civilian oversight investigations, risk of criminal liability, job loss). Therefore, we caution that no scenario-based study can truly replicate the naturalistic police environment or officer performance within it.

The current assessment of performance was also based on a single snapshot in time with one sample of officers from a specific agency. Consequently, the results may not generalize to other scenarios, other officers, or other agencies. On average, officers from the participating agency receive in-service training that aligns with other LEAs (i.e., 40 h annually; Reaves, 2010), their pre-service and supervised field training are significantly longer (i.e., 6 months each). Additionally, the agency is known to provide high quality training according to industry standards, as exemplified by its dedicated teams of experienced and expert learning designers (civilian and police), standardized training, centralized oversight of instructor training, and collaboration with academics to embed best training practices. Therefore, the results of this study may be reflective of performance with above-average quality training.

Lastly, measurements of HRV can be influenced by respiration and physical activity, which may obscure linkages between psychological and physiological processes (Laborde et al., 2017). However, to increase confidence in the study findings, the current study used measures which are relatively free of respiratory influences, reported baseline measures, used a scenario room with confined space to restrict movement, and followed recently proposed HRV reporting guidelines (Quintana et al., 2016; Laborde et al., 2017). As cardiovascular stress reactivity is only one aspect of the stress response system, future research should include as much biological sampling (e.g., HPA activity, blood markers) as is logistically and ethically possible (Supplementary Material L (https://osf.io/egkf6/)).

## Conclusion

Based on the robust methodology and relatively large sample of active-duty police officers used in this study, the results provide important insights into the general relationships between stress, training, experience, and performance in critical police incidents. The findings provide LEAs the opportunity to critically reflect on current training practices and offers a roadmap for making evidence-based improvements to training. The results also provide important evidence which may inform the reasonableness standard used in courts of law and paint a more realistic picture of police performance under stress given the current training available to officers. However, perhaps most importantly, we identify a need for a concerted effort to increase police training standards and ensure the necessary infrastructure is in place to achieve them. In this way, we should be able to enhance police performance in stressful police–citizen encounters and significantly reduce critical lethal force errors.

## Data Availability Statement

The datasets presented in this article are not readily available because of privacy and ethical restrictions. Requests to access the datasets should be directed to SB (simonbaldwin@cmail.carleton.ca).

## Ethics Statement

The studies involving human participants were reviewed and approved by the Carleton University Ethics Committee for Psychological Research (CUREB-B Clearance # 108733), as well as the Research Review Board (2018-04) of the agency from which the officers were recruited. The patients/participants provided their written informed consent to participate in this study. Written informed consent was obtained from the individual(s) for the publication of any potentially identifiable images or data included in this article.

## Author Contributions

SB, CB, and BB conceptualized the study and JA advised on stress measures during conceptualization. SB, BB, AB, BJ, CL, HM, and TS completed the data collection. SB performed the data analysis and interpretation with guidance from JA and under the supervision of CB. SB drafted the manuscript. CB, JA, BB, AB, BJ, CL, HM, and TS provided critical revisions. All authors contributed to the article and approved the submitted version.

## Funding

This research was funded by a Social Sciences and Humanities Research Council Insight grant awarded to CB (SSHRC# 435-2017-1354). The participating agency also provided funds to facilitate data collection, along with in-kind contributions to support the research.

## Author Disclaimer

The views expressed in the submitted article are the authors’ and not an official position of Carleton University, the University of Toronto, or the participating agency.

## Conflict of Interest

The authors declare that the research was conducted in the absence of any commercial or financial relationships that could be construed as a potential conflict of interest.

## Publisher’s Note

All claims expressed in this article are solely those of the authors and do not necessarily represent those of their affiliated organizations, or those of the publisher, the editors and the reviewers. Any product that may be evaluated in this article, or claim that may be made by its manufacturer, is not guaranteed or endorsed by the publisher.

## Footnotes

^1^Pursuant to an occupational health assessment, the individual is considered mentally and physically fit to perform the tasks and duties of a police officer. As this was not a diagnostic clinical study, we did not perform medical or psychiatric examinations of participants, however, we did examine self-reported cardiovascular disease (*n* = 4) and being on medication that affects HR (*n* = 10) in relation to the data. Several cardiovascular measures did significantly differ for the 10 participants who reported being on medication that affects HR, although they did not remain significant once Bonferroni corrected (*α* = 0.05/6 = 0.008). Out of an abundance of caution, all analysis involving stress reactivity was conducted with and without participants who reported being on medication that affects HR. An examination of the results did not demonstrate a difference in level of significance or effect size. Thus, all participants were retained in the study.

^2^To reduce the likelihood of lost or corrupted data, participants were also equipped with a Â©FirstBeat Bodyguard 2 Heart Rate Monitor (Firstbeat Technologies Ltd., Jyväskylä, Finland), which has also been validated against ECGs (Parak and Korhonen, 2013; Bogdány et al., 2016; Hinde et al., 2021). Data from the FirstBeat Bodyguard 2 were used to supplement HR and HRV data for 9% (*n* = 11) of participants.

^3^StressVestÂ® does not require the use of personal protective equipment (PPE) that is typically required when training with non-lethal training ammunition (aside from range certified eye protection). This allows participants to observe facial expressions, and does not restrict peripheral vision, nor does it impede communication, hearing, or movement. SecuriBlanksÂ® are used to maintain the fidelity of the firearm (i.e., recoil and percussion). The StressVestÂ® can be concealed under clothing, which avoids priming the participant (i.e., actor wearing bulky PPE) and improves decision-making by safely and flexibly enabling more actors to participate in the scenario.

## Supplementary Material

SUPPLEMENTARY MATERIAL INDEX https://doi.org/10.17605/OSF.IO/PKJNV.

## References

[ref1] Akinola M. Mendes W. B. (2012). Stress-induced cortisol facilitates threat-related decision making among police officers. Behav. Neurosci. 126, 167–174. doi: 10.1037/a0026657, PMID: 22141468

[ref2] Alison L. Crego J. (2012). Policing Critical Incidents: Leadership and Critical Incident Management. United Kingdom: Routledge.

[ref3] Alpert G. P. Smith W. C. (1994). How reasonable is the reasonable man? Police and excessive force. J. Crim. Law Criminol. 85, 481–501. doi: 10.2307/1144107

[ref4] Andersen J. P. Di Nota P. M. Beston B. Boychuk E. C. Gustafsberg H. Poplawski S.. (2018). Reducing lethal force errors by modulating police physiology. J. Occup. Environ. Med. 60, 867–874. doi: 10.1097/jom.0000000000001401, PMID: 30020222 PMC6200377

[ref5] Andersen J. P. Gustafsberg H. (2016). A training method to improve police use of force decision making. SAGE Open 6, 215824401663870. doi: 10.1177/2158244016638708

[ref6] Andersen J. P. Boychuk E. Di Nota P. M. Back D. Poplawski S. (2018). "Decision Model for Police Encounters: A Science-Based Approach for Decision Making in Police Encounters". (Toronto, Canada: University of Toronto Mississauga).

[ref7] Andersen J. P. Pitel M. Weerasinghe A. Papazoglou K. (2016). Highly realistic scenario based training simulates the psychophysiology of real world use of force encounters: implications for improved police officer performance. J. Law Enforc. 5

[ref8] Anderson G. S. Di Nota P. M. Metz G. A. S. Andersen J. P. (2019). The impact of acute stress physiology on skilled motor performance: implications for policing. Front. Psychol. 10:2501. doi: 10.3389/fpsyg.2019.02501, PMID: 31781001 PMC6856650

[ref9] Anderson G. Litzenberger R. Plecas D. (2002). Physical evidence of police officer stress. Policing 25, 399–420. doi: 10.1108/13639510210429437

[ref10] Anderson G. Plecas D. (2000). Predicting shooting scores from physical performance data. Policing 23, 525–537. doi: 10.1108/13639510010355611

[ref11] Anshel M. H. (2000). A conceptual model and implications for coping with stressful events in police work. Crim. Justice Behav. 27, 375–400. doi: 10.1177/0093854800027003006

[ref12] Anshel M. H. Robertson M. Caputi P. (1997). Sources of acute stress and their appraisals and reappraisals among Australian police as a function of previous experience. J. Occup. Organ. Psychol. 70, 337–356. doi: 10.1111/j.2044-8325.1997.tb00653.x

[ref13] Appelhans B. M. Luecken L. J. (2006). Heart rate variability as an index of regulated emotional responding. Rev. Gen. Psychol. 10, 229–240. doi: 10.1037/1089-2680.10.3.229

[ref14] Arble E. Daugherty A. M. Arnetz B. (2019). Differential effects of physiological arousal following acute stress on police officer performance in a simulated critical incident. Front. Psychol. 10:759. doi: 10.3389/fpsyg.2019.00759, PMID: 31024398 PMC6465322

[ref15] Arnetz B. B. Nevedal D. C. Lumley M. A. Backman L. Lublin A. (2009). Trauma resilience training for police: psychological and performance effects. J. Police Crim. Psychol. 24, 1–9. doi: 10.1007/s11896-008-9030-y

[ref16] Artwohl A. (2002). Perceptual and memory distortion during officer-involved shootings. FBI Law Enforc. Bull. 71, 18–24.

[ref17] Artwohl A. (2008). Perceptual and memory distortion during officer-involved shootings. FBI Law Enforc. Bull. 71, 18–24.

[ref18] Bakeman R. Goodman S. H. (2020). Interobserver reliability in clinical research: current issues and discussion of how to establish best practices. J. Abnorm. Psychol. 129, 5–13. doi: 10.1037/abn0000487, PMID: 31868383

[ref19] Baldwin S. Bennell C. Andersen J. P. Semple T. Jenkins B. (2019). Stress-activity mapping: physiological responses during general duty police encounters. Front. Psychol. 10:2216. doi: 10.3389/fpsyg.2019.02216, PMID: 31636582 PMC6788355

[ref20] Baldwin S. Blaskovits B. Widdershoven N. Marchant S. (2020). "2010 to 2019 Police Intervention Options Report". (Ottawa: Royal Canadian Mounted Police).

[ref21] Belur J. Agnew-Pauley W. Mcginley B. Tompson L. (2019). A systematic review of police recruit training programmes. Policing. 14, 76–90. doi: 10.1093/police/paz022

[ref22] Bennell C. Alpert G. Andersen J. P. Arpaia J. Huhta J. M. Kahn K. B.. (2021a). Advancing police use of force research and practice: urgent issues and prospects. Leg. Criminol. Psychol. 26, 121–144. doi: 10.1111/lcrp.12191

[ref23] Bennell C. Blaskovits B. Jenkins B. Semple T. Khanizadeh A.-J. Brown A. S.. (2021b). Promising practices for de-escalation and use-of-force training in the police setting: a narrative review. Policing 44, 377–404. doi: 10.1108/PIJPSM-06-2020-0092

[ref24] Berntson G. G. Cacioppo J. T. (2004). “Heart rate variability: stress and psychiatric conditions” in Dynamic Electrocardiography. eds. Marek M. John Camm A. (Hoboken, New Jersey: Blackwell Publishing), 57–64.

[ref25] Bertilsson J. Niehorster D. C. Fredriksson P. J. Dahl M. Granér S. Fredriksson O.. (2019). Stress levels escalate when repeatedly performing tasks involving threats. Front. Psychol. 10:1562. doi: 10.3389/fpsyg.2019.01562, PMID: 31333556 PMC6621421

[ref26] Blumberg D. M. Schlosser M. D. Papazoglou K. Creighton S. Kaye C. C. (2019). New directions in police academy training: a call to action. Int. J. Environ. Res. Public Health 16:4941. doi: 10.3390/ijerph16244941, PMID: 31817578 PMC6950698

[ref27] Bogdány T. Boros S. Szemerszky R. Köteles F. (2016). Validation of the firstbeat teambelt and bodyguard2 systems. Magy. Sporttud. Szle. 17, 5–12.

[ref28] Boulton L. Cole J. (2016). Adaptive flexibility: examining the role of expertise in the decision making of authorized firearms officers during armed confrontation. J. Cogn. Eng. Decis. Mak. 10, 291–308. doi: 10.1177/1555343416646684

[ref29] Brisinda D. Venuti A. Cataldi C. Efremov K. Iantorno E. Fenici R. (2015). Real-time imaging of stress-induced cardiac autonomic adaptation during realistic force-on-force police scenarios. J. Police Crim. Psychology 30, 71–86. doi: 10.1007/s11896-014-9142-5

[ref30] Brown A. Baldwin S. Blaskovits B. Bennell C. (2021). Examining the impact of grip strength and officer gender on shooting performance. Appl. Ergon. 97:103536. doi: 10.1016/j.apergo.2021.103536, PMID: 34364130

[ref31] Caminal P. Sola F. Gomis P. Guasch E. Perera A. Soriano N.. (2018). Validity of the polar V800 monitor for measuring heart rate variability in mountain running route conditions. Eur. J. Appl. Physiol. 118, 669–677. doi: 10.1007/s00421-018-3808-0, PMID: 29356949

[ref32] Castaldo R. Melillo P. Bracale U. Caserta M. Triassi M. Pecchia L. (2015). Acute mental stress assessment via short term HRV analysis in healthy adults: a systematic review with meta-analysis. Biomed. Signal Process. Control 18, 370–377. doi: 10.1016/j.bspc.2015.02.012

[ref33] Chan J. Andersen J. P. (2020). “Physiological stress responses associated with high-risk occupational duties” in Occupational Health. ed. Kavitha P. (London, United Kingdom: IntechOpen).

[ref34] Chrousos G. P. (2009). Stress and disorders of the stress system. Nat. Rev. Endocrinol. 5, 374–381. doi: 10.1038/nrendo.2009.106 19488073

[ref300] Cicchetti D. V. (1994). Guidelines, Criteria, and Rules of Thumb for Evaluating Normed and Standardized Assessment Instruments in Psychology. Psychological Assessment, 6, 284–290. doi: 10.1037/1040-3590.6.4.284

[ref35] Cilhoroz B. Giles D. Zaleski A. Taylor B. Fernhall B. Pescatello L. (2020). Validation of the polar V800 heart rate monitor and comparison of artifact correction methods among adults with hypertension. PLoS One 15:e0240220. doi: 10.1371/journal.pone.0240220, PMID: 33031480 PMC7544136

[ref36] Colin L. Nieuwenhuys A. Visser A. Oudejans R. R. D. (2014). Positive effects of imagery on police officers' shooting performance under threat. Appl. Cogn. Psychol. 28, 115–121. doi: 10.1002/acp.2972

[ref37] Condon C. C. (2015). Simulated Armed Confrontation and Police Decision Making: Examining the Impact of Psychophysiological Demand on Executive Functioning. Liverpool, United Kingdom: University of Liverpool.

[ref38] Criminal Code (1985). “Criminal Code, R.S.C. c. C-46.” (Canada). Available at: https://laws-lois.justice.gc.ca/eng/acts/c-46/

[ref39] Cyr K. (2016). Police use of force: assessing necessity and proportionality. Alta. Law Rev. 53:663. doi: 10.29173/alr424

[ref40] Cyr K. Ricciardelli R. Spencer D. (2020). Militarization of police: a comparison of police paramilitary units in Canadian and the United States. Int. J. Police Sci. Manag. 22, 137–147. doi: 10.1177/1461355719898204

[ref41] De Kloet E. R. Vreugdenhil E. Oitzl M. S. JoëLs M. (1998). Brain corticosteroid receptor balance in health and disease*. Endocr. Rev. 19, 269–301. doi: 10.1210/edrv.19.3.0331, PMID: 9626555

[ref42] Di Nota P. M. Andersen J. P. Huhta J.-M. Gustafsberg H. (2021a). “Evidence-based instruction of police use of force” in Interventions, Training, and Technologies for Improved Police Well-Being and Performance. eds. Arble E. Arnetz B. (Hershey Pennsylvania, USA: IGI Global), 72–101.

[ref43] Di Nota P. M. Arpaia J. Boychuk E. C. Collins P. Andersen Judith P. (2021b). Testing the efficacy of a 1-day police decision-making and autonomic modulation intervention: a quasi-random pragmatic trial. Front. Psychol. 12:719046. doi: 10.3389/fpsyg.2021.719046, PMID: 34456827 PMC8385198

[ref44] Di Nota P. M. Chan J. F. Huhta J.-M. Andersen J. P. (2021c). Considering objective and subjective measures for police use of force evaluation. Int. J. Environ. Res. Public Health 18:5351. doi: 10.3390/ijerph18105351, PMID: 34069786 PMC8157287

[ref45] Di Nota P. M. Huhta J.-M. (2019). Complex motor learning and police training: applied, cognitive, and clinical perspectives. Front. Psychol. 10:1797. doi: 10.3389/fpsyg.2019.01797, PMID: 31440184 PMC6692711

[ref46] Di Nota P. M. Stoliker B. E. Vaughan A. D. Andersen J. P. Anderson G. S. (2020). Stress and memory: a systematic state-of-the-art review with evidence-gathering recommendations for police. Policing 44, 1–17. doi: 10.1108/PIJPSM-06-2020-0093

[ref47] Donner C. M. Popovich N. (2018). Hitting (or missing) the mark: an examination of police shooting accuracy in officer-involved shooting incidents. Policing 42, 474–489. doi: 10.1108/PIJPSM-05-2018-0060

[ref48] Driskell J. E. Johnston J. H. (1998). “Stress exposure training” in Making Decisions Under Stress: Implications for Individual and Team Training. eds. Cannon-Bowers J. A. Salas E. (Washington, DC: American Psychological Association), 191–217.

[ref49] Driskell J. E. Johnston J. H. Salas E. (2001). Does stress training generalize to novel settings? Hum. Factors 43, 99–110. doi: 10.1518/001872001775992471, PMID: 11474766

[ref50] Driskell J. E. Salas E. (eds.). (1996). Stress and Human Performance. New Jersey, US: Lawrence Erlbaum Associates Inc.

[ref51] DuCharme S. D. (2002). The search for reasonableness in use-of-force cases: understanding the effects of stress on perception and performance. Fordham Law Rev. 70:2515

[ref52] Easterbrook J. A. (1959). The effect of emotion on cue utilization and the organization of behavior. Psychol. Rev. 66, 183–201. doi: 10.1037/h0047707, PMID: 13658305

[ref53] Engel R. S. Corsaro N. Isaza G. T. McManus H. D. (2020). Examining the Impact of Integrating Communications, Assessment, and Tactics (ICAT) de-Escalation Training for the Louisville Metro Police Department: Initial Findings. Center for Police Research and Policy.

[ref54] Engel R. S. Smith M. R. (2009). Perceptual distortion and reasonableness during police shootings: law, legitimacy, and future research. Criminol. Public Policy 8, 141–151. doi: 10.1111/j.1745-9133.2009.00538.x

[ref55] Eysenck M. W. Derakshan N. Santos R. Calvo M. G. (2007). Anxiety and cognitive performance: attentional control theory. Emotion 7, 336–353. doi: 10.1037/1528-3542.7.2.336 17516812

[ref56] Fagan J. Campbell A. (2020). Race and reasonableness in police killings. Boston Univ. Law Rev. 100, 951–1016.

[ref57] Faul F. Erdfelder E. Lang A.-G. Buchner A. (2007). G*power 3: a flexible statistical power analysis program for the social, behavioral, and biomedical sciences. Behav. Res. Methods 39, 175–191. doi: 10.3758/bf03193146, PMID: 17695343

[ref58] Fenici R. Brisinda D. Sorbo A. R. (2011). “Methods for real-time assessment of operational stress during realistic police tactical training” in Handbook of Police Psychology. ed. Kitaeff J. (Florence, US: Routledge), 295.

[ref59] Folkman S. Lazarus R. S. Dunkel-Schetter C. DeLongis A. Gruen R. J. (1986). Dynamics of a stressful encounter: cognitive appraisal, coping, and encounter outcomes. J. Pers. Soc. Psychol. 50, 992–1003. doi: 10.1037/0022-3514.50.5.992, PMID: 3712234

[ref60] Fridman J. Barrett L. F. Wormwood J. B. Quigley K. S. (2019). Applying the theory of constructed emotion to police decision making. Front. Psychol. 10:1946. doi: 10.3389/fpsyg.2019.01946, PMID: 31572250 PMC6749088

[ref61] Giessing L. Frenkel M. O. Zinner C. Rummel J. Nieuwenhuys A. Kasperk C.. (2019). Effects of coping-related traits and psychophysiological stress responses on police recruits’ shooting behavior in reality-based scenarios. Front. Psychol. 10:1523. doi: 10.3389/fpsyg.2019.01523, PMID: 31333547 PMC6617500

[ref62] Gilgen-Ammann R. Schweizer T. Wyss T. (2019). RR interval signal quality of a heart rate monitor and an ECG Holter at rest and during exercise. Eur. J. Appl. Physiol. 119, 1525–1532. doi: 10.1007/s00421-019-04142-5, PMID: 31004219

[ref63] Giuseppe G. Antonio B. Luigi Isaia L. Nicola M. Marco P. Mario N.. (2021). HRV in active-duty special forces and public order military personnel. Sustain. For. 13:3867. doi: 10.3390/su13073867

[ref64] Gröpel P. Mesagno C. (2019). Choking interventions in sports: a systematic review. Int. Rev. Sport Exerc. Psychol. 12, 176–201. doi: 10.1080/1750984x.2017.1408134

[ref65] Hernández-Vicente A. Hernando D. Marín-Puyalto J. Vicente-Rodríguez G. Garatachea N. Pueyo E.. (2021). Validity of the polar H7 heart rate sensor for heart rate variability analysis during exercise in different age, body composition and fitness level groups. Sensors 21:902. doi: 10.3390/s21030902, PMID: 33572800 PMC7866245

[ref66] Hinde K. White G. Armstrong N. (2021). Wearable devices suitable for monitoring twenty four hour heart rate variability in military populations. Sensors 21:1061. doi: 10.3390/s21041061, PMID: 33557190 PMC7913967

[ref67] Honig A. L. Lewinski W. J. (2008). A survey of the research on human factors related to lethal force encounters: implications for law enforcement training, tactics, and testimony. Law Enforc. Exec. Forum. 4, 129–152.

[ref68] Honig A. L. Sultan S. E. (2004). “Reactions and resilience under fire: what an officer can expect” in Police Chief, *vol*. 71, 54–60.

[ref69] Hope L. (2016). Evaluating the effects of stress and fatigue on police officer response and recall: a challenge for research, training, practice and policy. J. Appl. Res. Mem. Cogn. 5, 239–245. doi: 10.1016/j.jarmac.2016.07.008

[ref70] Hope L. Blocksidge D. Gabbert F. Sauer J. D. Lewinski W. J. Mirashi A.. (2016). Memory and the operational witness: police officer recall of firearms encounters as a function of active response role. Law Hum. Behav. 40, 23–35. doi: 10.1037/lhb0000159, PMID: 26436335

[ref71] IBM Corp (2020). IBM SPSS Statistics for Windows, Version 27.0. (Armonk, NY: IBM Corp).

[ref72] International Association of Chiefs of Police (2020). National Consensus Policy and Discussion Paper on Use of Force. International Association of Chiefs of Police).

[ref73] James L. Goldstein Michael S. Lecy P. Mase S. (2020). Testing the impact of physiological stress response on police performance during critical job tasks. Policing 44, 405–417. doi: 10.1108/PIJPSM-04-2020-0060

[ref74] James L. James S. Davis R. Dotson E. (2019). Using interval-level metrics to investigate situational-, suspect-, and officer-level predictors of police performance during encounters with the public. Police Q. 22, 452–480. doi: 10.1177/1098611119857559

[ref75] Jenkins B. Semple T. Quail J. Bennell C. (2021). “Optimizing scenario-based training for law enforcement” in Interventions, Training, and Technologies for Improved Police Well-Being and Performance. eds. Arble E. Arnetz B. (Hershey, Pennsylvania, USA: IGI Global), 18–37.

[ref76] Johnson R. R. Stone B. T. Miranda C. M. Vila B. James L. James S. M.. (2014). Identifying psychophysiological indices of expert vs. novice performance in deadly force judgment and decision making. Front. Hum. Neurosci. 8:512. doi: 10.3389/fnhum.2014.00512, PMID: 25100966 PMC4107851

[ref77] Johnston J. H. Driskell J. E. Salas E. (1997). Vigilant and hypervigilant decision making. J. Appl. Psychol. 82, 614–622. doi: 10.1037/0021-9010.82.4.614, PMID: 9378686

[ref78] Kahneman D. Klein G. (2009). Conditions for intuitive expertise: a failure to disagree. Am. Psychol. 64, 515–526. doi: 10.1037/a0016755, PMID: 19739881

[ref79] Kalisch R. Müller M. B. Tüscher O. (2015). A conceptual framework for the neurobiological study of resilience. Behav. Brain Sci. 38:e92. doi: 10.1017/s0140525x1400082x, PMID: 25158686

[ref80] Kavanagh J. (2005). "Stress and Performance: A Review of the Literature and its Applicability to the Military". (Santa Monica, California: RAND Corporation).

[ref81] Kavanagh E. L. (2006). A cognitive model of firearms policing. J. Police Crim. Psychol. 21, 25–36. doi: 10.1007/BF02855682

[ref82] Kelley D. C. Siegel E. Wormwood J. B. (2019). Understanding police performance under stress: insights from the biopsychosocial model of challenge and threat. Front. Psychol. 10:1800. doi: 10.3389/fpsyg.2019.01800, PMID: 31447738 PMC6696903

[ref83] Kemeny M. E. (2003). The psychobiology of stress. Curr. Dir. Psychol. Sci. 12, 124–129. doi: 10.1111/1467-8721.01246

[ref84] Kent S. Devonport T. J. Lane A. M. Nicholls W. Friesen A. P. (2018). The effects of coping interventions on ability to perform under pressure. J. Sports Sci. Med. 17, 40–55. PMID: 29535577 PMC5844208

[ref85] Klein G. (1997). “The recognition-primed decision (RPD) model: looking back, looking forward” in Naturalistic Decision Making. eds. Klein G. Zsambok C. E. (Hillsdale, NJ, US: Lawrence Erlbaum Associates, Inc.), 285–292.

[ref86] Klein G. (1999). Sources of Power: How People Make Decisions. Cambridge, Mass: The MIT Press.

[ref87] Klein G. (2015). Reflections on applications of naturalistic decision making. J. Occup. Organ. Psychol. 88, 382–386. doi: 10.1111/joop.12122 PMC6020850 29962664

[ref88] Klein G. Borders J. (2016). The shadowbox approach to cognitive skills training: an empirical evaluation. J. Cogn. Eng. Decis. Mak. 10, 268–280. doi: 10.1177/1555343416636515

[ref89] Klinger D. Brunson R. (2009). Police officers' perceptual distortions during lethal force situations: informing the reasonableness standard. Criminol. Public Policy. 8, 117–140. doi: 10.1111/j.1745-9133.2009.00537.x

[ref90] Koo T. K. Li M. Y. (2016). A guideline of selecting and reporting intraclass correlation coefficients for reliability research. J. Chiropr. Med. 15, 155–163. doi: 10.1016/j.jcm.2016.02.012, PMID: 27330520 PMC4913118

[ref91] Laborde S. Mosley E. Thayer J. F. (2017). Heart rate variability and cardiac vagal tone in psychophysiological research – recommendations for experiment planning, data analysis, and data reporting. Front. Psychol. 8:213. doi: 10.3389/fpsyg.2017.00213, PMID: 28265249 PMC5316555

[ref92] Landman A. Nieuwenhuys A. Oudejans R. R. D. (2016a). Decision-related action orientation predicts police officers' shooting performance under pressure. Anxiety Stress Coping 29, 570–579. doi: 10.1080/10615806.2015.1070834, PMID: 26215425

[ref93] Landman A. Nieuwenhuys A. Oudejans R. R. D. (2016b). The impact of personality traits and professional experience on police officers' shooting performance under pressure. Ergonomics 59, 950–961. doi: 10.1080/00140139.2015.1107625, PMID: 26467525

[ref94] LeDoux J. E. Pine D. S. (2016). Using neuroscience to help understand fear and anxiety: a two-system framework. Am. J. Psychiatr. 173, 1083–1093. doi: 10.1176/appi.ajp.2016.16030353, PMID: 27609244

[ref95] Leger (2021). Police Reputation: 2020 Community Update. (Canada). Available at: https://cacp.ca/index.html?asst_id=2494

[ref96] Lovallo W. R. (2016). Stress & Health: Biological and Psychological Interactions. Los Angeles: SAGE.

[ref97] Low W. R. Sandercock G. R. H. Freeman P. Winter M. E. Butt J. Maynard I. (2021). Pressure training for performance domains: a meta-analysis. Sport Exerc. Perform. Psychol. 10, 149–163. doi: 10.1037/spy0000202

[ref98] Lundell R. V. Tuominen L. Ojanen T. Parkkola K. Räisänen-Sokolowski A. (2021). Diving responses in experienced rebreather divers: short-term heart rate variability in cold water diving. Front. Physiol. 12:649319. doi: 10.3389/fphys.2021.649319, PMID: 33897457 PMC8058382

[ref99] Mangels L. Suss J. Lande B. (2020). Police expertise and use of force: using a mixed-methods approach to model expert and novice use-of-force decision-making. J. Police Crim. Psychol. 35, 294–303. doi: 10.1007/s11896-020-09364-4

[ref100] McCraty R. Atkinson M. (2012). Resilience training program reduces physiological and psychological stress in police officers. Glob. Adv. Health Med. 1, 44–66. doi: 10.7453/gahmj.2012.1.5.013, PMID: 27257532 PMC4890098

[ref101] McEwen B. S. (1998). Stress, adaptation, and disease. Allostasis and allostatic load. Ann. N. Y. Acad. Sci. 840, 33–44. doi: 10.1111/j.1749-6632.1998.tb09546.x 9629234

[ref102] McLachlin B. Binnie W. I. C. LeBel L. Deschamps M. Fish M. J. Abella R. S.. (2010). “R. v. Nasogaluak, 2010 SCC 6, [2010] 1 S.C.R. 206.” Case Law. Available at: https://scc-csc.lexum.com/scc-csc/scc-csc/en/item/7845/index.do.

[ref103] Meyerhoff J. L. Norris W. Saviolakis G. A. Wollert T. Burge B. O. B. Atkins V.. (2004). Evaluating performance of law enforcement personnel during a stressful training scenario. Ann. N. Y. Acad. Sci. 1032, 250–253. doi: 10.1196/annals.1314.031, PMID: 15677421

[ref104] Miles-Chan J. L. Sarafian D. Montani J. P. Schutz Y. Dulloo A. G. (2013). Sitting comfortably versus lying down: is there really a difference in energy expenditure? Clin. Nutr. 33, 175–178. doi: 10.1016/j.clnu.2013.11.009, PMID: 24290343

[ref105] Morrison G. B. Garner T. K. (2011). Latitude in deadly force training: progress or problem? Police Pract. Res. 12, 341–361. doi: 10.1080/15614263.2011.563968

[ref106] Morrison G. B. Vila B. J. (1998). Police handgun qualification: practical measure or aimless activity? Policing 21, 510–533. doi: 10.1108/13639519810228804

[ref107] Mugford R. Corey S. Bennell C. (2013). Improving police training from a cognitive load perspective. Policing 36, 312–337. doi: 10.1108/13639511311329723

[ref108] Nieuwenhuys A. Oudejans R. R. D. (2010). Effects of anxiety on handgun shooting behavior of police officers: a pilot study. Anxiety Stress Coping 23, 225–233. doi: 10.1080/10615800902977494, PMID: 19462309

[ref109] Nieuwenhuys A. Oudejans R. R. D. (2011a). Anxiety and perceptual-motor performance: toward an integrated model of concepts, mechanisms, and processes. Psychol. Res. 76, 747–759. doi: 10.1007/s00426-011-0384-x 22038472 PMC3470682

[ref110] Nieuwenhuys A. Oudejans R. R. D. (2011b). Training with anxiety: short- and long-term effects on police officers’ shooting behavior under pressure. Cogn. Process. 12, 277–288. doi: 10.1007/s10339-011-0396-x, PMID: 21431863 PMC3142543

[ref111] Nieuwenhuys A. Oudejans R. R. (2017). Anxiety and performance: perceptual-motor behavior in high-pressure contexts. Curr. Opin. Psychol. 16, 28–33. doi: 10.1016/j.copsyc.2017.03.019, PMID: 28813350

[ref112] Nieuwenhuys A. Savelsbergh G. J. P. Oudejans R. R. D. (2012). Shoot or don't shoot? Why police officers are more inclined to shoot when they are anxious. Emotion 12, 827–833. doi: 10.1037/a0025699 22023363

[ref113] Nieuwenhuys A. Savelsbergh G. J. P. Oudejans R. R. D. (2015). Persistence of threat-induced errors in police officers' shooting decisions. Appl. Ergon. 48, 263–272. doi: 10.1016/j.apergo.2014.12.006, PMID: 25683553

[ref114] Nieuwenhuys A. Weber J. van der Hoeve R. Oudejans R. R. D. (2016). Sitting duck or scaredy-cat? Effects of shot execution strategy on anxiety and police officers’ shooting performance under high threat. Leg. Criminol. Psychol. 22, 274–287. doi: 10.1111/lcrp.12099

[ref115] O’Neill J. O’Neill D. A. Weed K. Hartman M. E. Spence W. Lewinski W. J. (2019). Police academy training, performance, and learning. Behav. Anal. Pract. 12, 353–372. doi: 10.1007/s40617-018-00317-2, PMID: 31976241 PMC6745755

[ref116] Pallant J. (2010). SPSS Survival Manual a Step by Step Guide to Data Analysis Using SPSS. Maidenhead: Open University Press/McGraw-Hill.

[ref117] Parak J. Korhonen I. (2013). Accuracy of Firstbeat Bodyguard 2 Beat-to-Beat Heart Rate Monitor. Tampere, Finland: White Pap Firstbeat Technol Ltd.

[ref118] Public Prosecution Service of Canada (2018). RCMP Sentenced in Labour Code Trial [Online]. Ottawa, Ontario: Government of Canada. Available at: https://www.ppsc-sppc.gc.ca/eng/nws-nvs/2018/26_01_18.html (Accessed August, 2021).

[ref119] Quintana D. S. Alvares G. A. Heathers J. A. J. (2016). Guidelines for reporting articles on psychiatry and heart rate variability (GRAPH): recommendations to advance research communication. Transl. Psychiatry 6:e803. doi: 10.1038/tp.2016.73, PMID: 27163204 PMC5070064

[ref120] Rajakaruna N. Henry P. J. Cutler A. Fairman G. (2017). Ensuring the validity of police use of force training. Police Pract. Res. 18, 507–521. doi: 10.1080/15614263.2016.1268959

[ref121] Reason J. (2000). Human error: models and management. Br. Med. J. 320, 768–770. doi: 10.1136/bmj.320.7237.768, PMID: 10720363 PMC1117770

[ref122] Reaves B. A. (2010). "Local Police Departments, 2007". (Washington, DC: Bureau of Justice Statistics).

[ref123] Reaves B. A. (2016). "State and Local Law Enforcement Training Academies, 2013". (Washington, DC: Bureau of Justice Statistics).

[ref124] Rehnquist W. (1989). “Graham v. Connor - 490 U.S. 386.” U.S. Supreme Court. Case law. Available at: https://supreme.justia.com/cases/federal/us/490/386/

[ref125] Renden P. G. Landman H. M. Daalder N. R. de Cock H. P. Savelsbergh G. J. P. Oudejans R. R. D. (2017). Effects of threat, trait anxiety and state anxiety on police officers' actions during an arrest. Leg. Criminol. Psychol. 22, 116–129. doi: 10.1111/lcrp.12077

[ref126] Renden P. G. Landman A. Geerts S. F. Jansen S. E. M. Faber G. S. Savelsbergh G. J. P.. (2014). Effects of anxiety on the execution of police arrest and self-defense skills. Anxiety Stress Coping 27, 100–112. doi: 10.1080/10615806.2013.810213, PMID: 23837827

[ref127] Renden P. G. Landman A. Savelsbergh G. J. P. Oudejans R. R. D. (2015). Police arrest and self-defence skills: performance under anxiety of officers with and without additional experience in martial arts. Ergonomics 58, 1496–1506. doi: 10.1080/00140139.2015.1013578, PMID: 25679517

[ref128] Rimmele U. Zellweger B. C. Marti B. Seiler R. Mohiyeddini C. Ehlert U.. (2007). Trained men show lower cortisol, heart rate and psychological responses to psychosocial stress compared with untrained men. Psychoneuroendocrinology 32, 627–635. doi: 10.1016/j.psyneuen.2007.04.005, PMID: 17560731

[ref129] Rojek J. Grieco J. Meade B. Parsons D. (2020). "National Survey on Officer Safety Training: Findings and Implications". (Washington, DC: National Police Foundation).

[ref130] Roos L. E. Knight E. L. Beauchamp K. G. Berkman E. T. Faraday K. Hyslop K.. (2017). Acute stress impairs inhibitory control based on individual differences in parasympathetic nervous system activity. Biol. Psychol. 125, 58–63. doi: 10.1016/j.biopsycho.2017.03.004, PMID: 28268165 PMC5448703

[ref131] Sahoo T. K. Mahapatra A. Ruban N. (2019). “Stress index calculation and analysis based on heart rate variability of ECG signal with arrhythmia,” in *2019 Innovations in Power and Advanced Computing Technologies (i-PACT). IEEE*.

[ref132] Sapolsky R. M. (2004). Why Zebras Don't Get Ulcers: The Acclaimed Guide to Stress, Stress-Related Diseases, and Coping. New York: Holt Paperbacks.

[ref133] Saunders T. Driskell J. E. Johnston J. H. Salas E. (1996). The effect of stress inoculation training on anxiety and performance. J. Occup. Health Psychol. 1, 170–186. doi: 10.1037/1076-8998.1.2.170, PMID: 9547044

[ref134] Saus E.-R. Johnsen B. H. Eid J. Riisem P. K. Andersen R. Thayer J. F. (2006). The effect of brief situational awareness training in a police shooting simulator: an experimental study. Mil. Psychol. 18, S3–S21. doi: 10.1207/s15327876mp1803s_2

[ref135] Singh I. (2020). 2020 Already a Particularly Deadly Year for People Killed in Police Encounters, CBC research shows. CBC News.

[ref136] Spangler D. P. Gamble K. R. McGinley J. J. Thayer J. F. Brooks J. R. (2018). Intra-individual variability in vagal control is associated with response inhibition under stress. Front. Hum. Neurosci. 12:475. doi: 10.3389/fnhum.2018.00475, PMID: 30542274 PMC6277930

[ref137] Staal M. A. (2004). Stress, Cognition, and Human Performance: A Literature Review and Conceptual Framework. Washington, DC: NASA.

[ref138] Suss J. Ward P. (2018). Revealing perceptual–cognitive expertise in law enforcement: an iterative approach using verbal-report, temporal-occlusion, and option-generation methods. Cogn. Tech. Work 20, 585–596. doi: 10.1007/s10111-018-0493-z

[ref139] Syed M. Nelson S. C. (2015). Guidelines for establishing reliability when coding narrative data. Emerg. Adulthood 3, 375–387. doi: 10.1177/2167696815587648

[ref140] Taverniers J. De Boeck P. (2014). Force-on-force handgun practice: an intra-individual exploration of stress effects, biomarker regulation, and behavioral changes. Hum. Factors 56, 403–413. doi: 10.1177/0018720813489148, PMID: 24689257

[ref141] Taylor P. L. (2019). Beyond false positives: a typology of police shooting errors. Criminol. Public Policy 18, 807–822. doi: 10.1111/1745-9133.12460

[ref142] Thayer J. F. Ahs F. Fredrikson M. Sollers J. J. Wager T. D. (2012). A meta-analysis of heart rate variability and neuroimaging studies: implications for heart rate variability as a marker of stress and health. Neurosci. Biobehav. Rev. 36, 747–756. doi: 10.1016/j.neubiorev.2011.11.009, PMID: 22178086

[ref143] Thayer J. F. Hansen A. L. Saus-Rose E. Johnsen B. H. (2009). Heart rate variability, prefrontal neural function, and cognitive performance: the neurovisceral integration perspective on self-regulation, adaptation, and health. Ann. Behav. Med. 37, 141–153. doi: 10.1007/s12160-009-9101-z, PMID: 19424767

[ref144] Thayer J. F. Sternberg E. (2006). Beyond heart rate variability: vagal regulation of allostatic systems. Ann. N. Y. Acad. Sci. 1088, 361–372. doi: 10.1196/annals.1366.014, PMID: 17192580

[ref145] Tsigos C. Chrousos G. P. (2002). Hypothalamic–pituitary–adrenal axis, neuroendocrine factors and stress. J. Psychosom. Res. 53, 865–871. doi: 10.1016/S0022-3999(02)00429-4 12377295

[ref146] Tsigos C. Kyrou I. Kassi E. Chrousos G. P. (2020). Stress: Endocrine Physiology and Pathophysiology. Endotext [Internet].

[ref147] Vickers J. N. (2007). Perception, Cognition, and Decision Training: The Quiet Eye in Action. Windsor, Canada: Human Kinetics.

[ref148] Vickers J. N. Lewinski W. J. (2012). Performing under pressure: gaze control, decision making and shooting performance of elite and rookie police officers. Hum. Mov. Sci. 31, 101–117. doi: 10.1016/j.humov.2011.04.004 21807433

[ref149] Vickers J. N. Williams A. M. (2007). Performing under pressure: the effects of physiological arousal, cognitive anxiety, and gaze control in biathlon. J. Mot. Behav. 39, 381–394. doi: 10.3200/jmbr.39.5.381-394, PMID: 17827115

[ref150] Vila B. James S. James L. (2018). How police officers perform in encounters with the public: measuring what matters at the individual level. Policing 41, 215–232. doi: 10.1108/PIJPSM-11-2016-0166

[ref151] Vine S. J. Moore L. J. Wilson M. R. (2016). An integrative framework of stress, attention, and visuomotor performance. Front. Psychol. 7:1671. doi: 10.3389/fpsyg.2016.01671, PMID: 27847484 PMC5088191

[ref152] Violanti J. M. (2014). Dying for the Job: Police Work Exposure and Health. Springfield, Illinois: Charles C. Thomas, Publisher, Ltd.

[ref153] Ward P. Suss J. Eccles D. W. Williams A. M. Harris K. R. (2011). Skill-based differences in option generation in a complex task: a verbal protocol analysis. Cogn. Process. 12, 289–300. doi: 10.1007/s10339-011-0397-9, PMID: 21461753

[ref154] Wollert T. Driskell J. E. Quail J. (2011). Stress Exposure Training Guidelines: Instructor Guide to Reality-Based Training. U.S.D.o.H. Security. Available at: http://www.virtualtacticalacademy.com/files/stress exposure training manual 9-26B.pdf. (Accessed March 20, 2017).

[ref155] Yerkes R. M. Dodson J. D. (1908). The relation of strength of stimulus to rapidity of habit-formation. J. Comp. Neurol. Psychol. 18, 459–482. doi: 10.1002/cne.920180503

[ref156] Zamoff M. (2020). Determining the perspective of a reasonable police officer: an evidence-based proposal. Villanova Law Rev. 65:585

