# Supplementary material for: A Reasonable Officer: Examining the Relationships Among Stress, Training, and Performance in a Highly Realistic Lethal Force Scenario
Source: Front Psychol. 2022 Jan 17;12:759132. doi: 10.3389/fpsyg.2021.759132 (PMC8803048; doi:10.3389/fpsyg.2021.759132)
Supplement: SUPPLEMENTARY MATERIAL INDEX — https://doi.org/10.17605/OSF.IO/PKJNV. [file Data_Sheet_1.zip › Supplementary Material A.pdf]

Supplementary Material A - Scenario Design

The scenario was developed by the authors, based on extensive reviews of the agency's officer-involved shootings and UoF encounters. This ensured the scenario was realistic and reflective of the operational environment. In order to increase the stress of the scenario, several psychological stressors from the literature were embedded in the scenario (Driskell and Salas, 1996; Wollert and Quail, 2018; Jenkins et al., 2020). These stressors included time pressure (i.e., a countdown in which the subject had a knife to their throat and indicated they would kill themselves), task load (e.g., multiple subjects), threat (e.g., a localized shock to the abdomen, if shot), ambiguity (e.g., situation inconsistent with dispatch information), novelty (e.g., the subject drawing a firearm after a knife had been discarded), role conflict (e.g., protecting bystander vs. de-escalating armed threat), noise (e.g., loud music playing, constant distractions from the bystander), performance pressure (e.g., instructor observing and video recording), distance (e.g., confined space), role ambiguity (e.g., providing medical attention immediately vs. waiting for emergency medical services [EMS]), and coordination demands (e.g. requesting backup, EMS).

Prior to the study, the scenario was pilot tested on a small sample of officers (N = 12) and then revised to ensure that it remained standardized despite there being multiple ways a participant could initially react.[1] The same two actors were present in every scenario. They followed a script that was flexible enough to ensure they could adapt to whatever decisions a participant made.

---

[1] Since the pilot was conducted in a different location than the study and the scenario was refined after the pilot, participants from the pilot were not included in the study sample.

The scenario occurred in a building that had been designed to appear as an apartment complex (see Figure 1) in a rural setting. The facilitator told the participant to communicate with them through their radio and respond to the call as they would in real-life. All participants were then dispatched to a second-floor apartment for a call from a female complainant indicating that her son (the subject) had been drinking heavily and was in breach of his probation conditions. The son refused to turn down the stereo and dispatch had difficulty hearing the complainant over the music. Information about the location and residence were provided to the participant. The nature of the call made it appropriate for a single officer to respond. At that point, the facilitator said, “scenario on” and, if they chose to do so, participants had the opportunity to ask dispatch for additional information. Given the rural environment, if backup was requested, the participant was advised that their only backup was currently on another call and unavailable. Responses for questions to dispatch were scripted for the facilitators to ensure consistency.

Figure 1

Experimental Setup of the Scenario

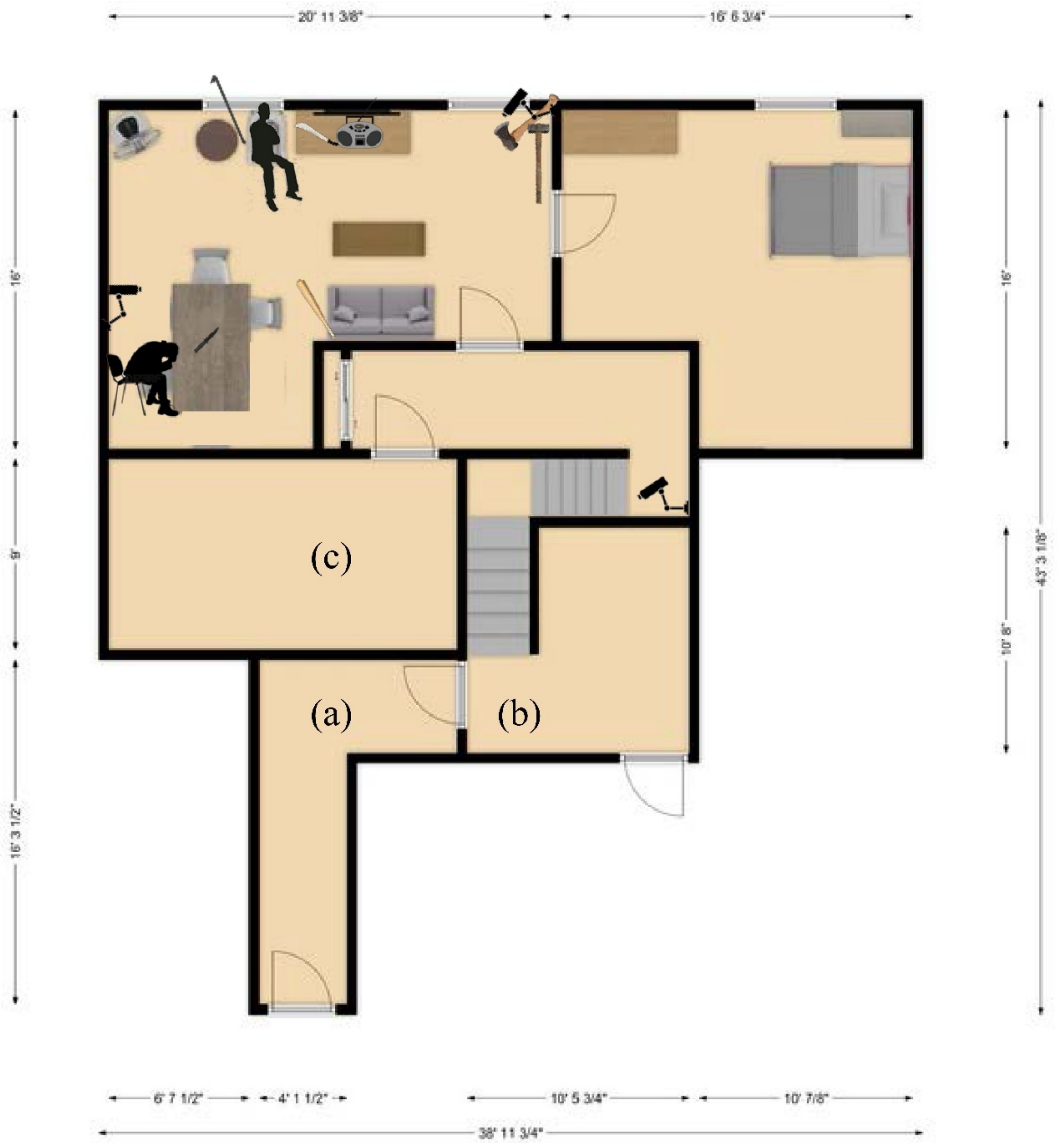

Note. Room measurements in feet/inches. (a) secondary safety inspection, equipping, and safety briefing area; (b) dispatch location; (c) out of bounds (bathroom).

During the approach to the second-floor apartment, the participants walked up 14 steps. Upon arriving "on scene" and knocking on the door of the residence, the participant was greeted by a white male (the bystander), who was the boyfriend of the subject (also white) and the actual

son of the female (mother) complainant. The music from a radio remained at a high volume. The disgruntled bystander agitatedly explained that the subject assaulted his mother (who had now left the residence) and wanted the subject removed from the residence. This changed the scenario from a breach of probation conditions to a domestic assault, providing the participant necessary grounds for arrest. Upon entering the apartment, there was a living/dining room (with a couch, coffee table, television, dining table, and other miscellaneous items) and a separate bedroom (complete with a bed, wardrobe, and clothes). There were several weapons (i.e., baseball bat, axe, sledgehammer, crowbar, machete) in plain sight throughout the apartment and a scent training system (AirAware®) released a marijuana-like smell.

The bystander immediately sat on a chair, offset between the entry and subject. The bystander refused to comply with any demands from the participant and continued to demand the participant to remove the subject, who was seated at the dining room table at the other end of the room. A partially obscured knife, within arms reach of the subject, was present on the dining room table among several empty bottles of alcohol. The confrontational subject remained seated at the table and refused to comply with directions from the participant. The subject eventually drew a knife and put it to his throat threatening to die by suicide. This provided the officers with the opportunity to attempt to de-escalate the situation and/or use intervention options. Any less-lethal intervention employed by the participant were feigned as being ineffective by the subject (e.g., swiping away conducted energy weapon probes, wiping away OC spray).

After some time passed, regardless of how much the officer attempted to verbally de-escalate or intervene, the subject complied and threw the knife on the ground towards the participant. The scenario was allowed to naturally unfold a little longer until the subject spontaneously pulled a firearm, stood up, and started to shoot at the participant, activating the

StressVest™ response (i.e., a localized shock to the abdomen).[2] This resulted in a lethal force response from the participant. Once shot at by the participant, the subject feigned a gunshot wound to the chest while the bystander contemporaneously produced and pointed a cellphone, verbally indicating that they were video recording the situation. From that point forward, the subject and the bystander were both compliant and followed any subsequent commands from the participant.

Participants were then provided the opportunity to prioritize and perform whatever actions they deemed necessary (e.g., request resources, secure weapons, physically restrain subject and/or bystander, search subject, administer first aid). To indicate some imminent action was required from the participant, both the subject and bystander referenced the subject's deteriorating condition from the gunshot wound and, if requested, dispatch relayed that back-up and EMS were 20 minutes away. The scenario was allowed to come to a natural conclusion and was ended by the facilitator when the participant failed to demonstrate any new actions or strategies. The scenario did not result in a fatal outcome. After the scenario, the scenario room was re-adjusted so that it appeared the same for each new participant.

.

[2] If the participant maintained a position of concealment (i.e., doorway at back for room) during the scenario, the bystander would walk over to the subject, who would then start choking the bystander. This behaviour would force the officer to move from their position of cover at which point, the subject would draw and shoot at the participant.

References

Driskell, J.E., and Salas, E. (eds.). (1996). *Stress and human performance.* New Jersey, US: Lawrence Erlbaum Associates Inc.

Jenkins, B., Semple, T., and Bennell, C. (2020). An evidence-based approach to critical incident scenario development. *Policing: An International Journal* ahead-of-print(ahead-of-print). doi: 10.1108/PIJPSM-02-2020-0017.

Wollert, T.N., and Quail, J. (2018). *A Scientific Approach to Reality Based Training.* Three Pistols Publishing.
