# Supplementary material for: A Reasonable Officer: Examining the Relationships Among Stress, Training, and Performance in a Highly Realistic Lethal Force Scenario
Source: Front Psychol. 2022 Jan 17;12:759132. doi: 10.3389/fpsyg.2021.759132 (PMC8803048; doi:10.3389/fpsyg.2021.759132)
Supplement: SUPPLEMENTARY MATERIAL INDEX — https://doi.org/10.17605/OSF.IO/PKJNV. [file Data_Sheet_1.zip › Supplementary Material B.pdf]

Supplementary Material B - Cardiovascular Reactivity Technical Specification

While there are strengths and limitations for all tools that measure stress reactivity (e.g., logistics and invasiveness of biological samples, time of day confounds with salivary cortisol), theoretical knowledge and empirical research support the use of heart rate variability (HRV) as one of the most precise non-invasive measure of psychological and physiological arousal (Berntson and Cacioppo, 2004; Appelhans and Luecken, 2006; Thayer et al., 2012). For example, a meta-analysis conducted by Thayer and colleagues (2012) found HRV to be associated with neural activation of areas involved in threat perception (i.e., the amygdala and medial prefrontal cortex). Stress reactivity is associated with an increase in sympathetic activation and suppression of parasympathetic activity (Malik et al., 1996; Berntson and Cacioppo, 2004; Castaldo et al., 2015). HRV captures the interplay between these two antagonistic systems (Appelhans and Luecken, 2006), and is regularly used as an objective physiological stress index (e.g., Haller et al., 2014; Giessing et al., 2019; James et al., 2020).

Heart rate (HR) and HRV were captured using monitoring devices. For logistical reasons, cardiovascular stress reactivity measures at rest in this study were based on the lowest one-minute HR, while sitting and completing pre-scenario paperwork for the study. Similar methods for determining cardiovascular reactivity at rest have been used in previous research (Anderson et al., 2002; Andersen and Gustafsberg, 2016; Baldwin et al., 2019). For comparative purposes, true resting cardiovascular reactivity during sleep was collected for a quarter of the sample (n = 29). HR_{mean} (bpm) and HR_{max} (bpm) represent the average and highest HR during each phase of the scenario, respectively.

Unlike analysis of absolute HR, HRV analysis is generally interested in how intervals change from beat to beat and by how much (Fenici et al., 2011; Tarvainen et al., 2016). The R

wave is the most prominent and easily detectable peak in the cardiac rhythm and as a result, is typically used to calculate the inter-beat-interval or “R-R interval” (Tarvainen et al., 2016). From R-R intervals, various time-domain measures can then be calculated that summarize a series of successive R-R interval values and thus the variability in HR (Tarvainen et al., 2016).

To examine HR and HRV, data were entered into ©Kubios HRV Premium Version 3.3.1. (Biomedical Signal Analysis Group, Department of Applied Physics, University of Kuopio, Finland), which is research software for the analysis of HRV. Samples were created for each phase of the scenario (e.g., dispatch, critical). Pre-processing of data in ©Kubios included automatic detection and correction of artifacts with interpolated beats (Lipponen and Tarvainen, 2019). Tarvainen and colleagues (2020) suggests correcting no more than 5% of beats to maintain data accuracy (e.g., avoid distortions), thus any HRV measure within a phase of the scenario that exceeded this threshold was removed. A detrending method (i.e., smoothness priors method) was used to remove very low frequency trend components for short-term HRV analysis (Tarvainen et al., 2020). The correlation between gold-standard five-minute recording length and the very-short-term intervals (i.e., 30s) used in this study has recently been reported and shown promise for police research, particularly during realistic police training (Smith et al., 2013; Brisinda et al., 2015; Munoz et al., 2015).

Reflective of factors indicative of PNS and SNS activity outlined in the literature (Malik et al., 1996; Berntson et al., 1997; Rajendra et al., 2006), the PNS Index and SNS Index, computed in Kubios HRV software, were used in this study. These or similar indices have been used in other research with samples of civilians (Ayuso-Moreno et al., 2020; Lundell et al., 2021), police (James et al., 2020) and special forces (Giuseppe et al., 2021). The PNS Index uses mean R-R intervals, root mean square of successive differences between normal heartbeats

(RMSSD), and normalized Poincaré plot index SD1, which is associated to RMSSD (Kubios Oy, 2021). Importantly, RMSSD reflects cardiac vagal tone, the contribution of the PNS to cardiac regulation, which is relatively free of respiratory influences (Laborde et al., 2017). Poincaré plots are also viewed as indicators of vagal activity and reduced cardiac vagal control, which are associated with both physiological and psychological strain and stress (Laborde et al., 2017). A PNS index value of zero indicates that the three parameters reflecting PNS activity are on average equal to the normal population average, while a positive or negative PNS index value indicates the number of SDs above or below the normal population average (Kubios Oy, 2021). During stress, much lower PNS index values can be expected.

The SNS index is computed using mean HR interval, normalized Poincaré plot index SD2, which is associated with standard deviation of normal to normal (R-R) intervals (SDNN), and the Baevsky Index of Regulatory System Tension or Stress Index (Kubios Oy, 2021). The Baevsky Stress Index is a geometric measure of HRV reflecting cardiovascular system stress (Baevsky, 2009). It is calculated based on a histogram distribution of R-R intervals (bin width 50 msec), using the following formula:

$$SI = \frac{\text{Amo x } 100\%}{\text{2Mo x MxDMn}}$$

Mo (mode) is the most frequently occurring R-R interval. The mode amplitude (AMo) is the count of the mode, presented as a percentage. MxDMn is the difference between minimum and maximum R-R intervals represents the amount of variation. Under psychological or physical stress the distribution of the histogram constricts, while simultaneously increasing in height (Korotkov, 2017). Therefore, high Stress Index values indicate reduced variability and increased SNS activation (Kubios Oy, 2021). The SNS index is interpreted similar to the PNS index, with values ranging as high as 5-35 during stress or high intensity exercise (Kubios Oy, 2021).

References

Andersen, J.P., and Gustafsberg, H. (2016). A Training Method to Improve Police Use of Force Decision Making. *SAGE Open* 6(2). doi: 10.1177/2158244016638708.

Anderson, G., Litzenberger, R., and Plecas, D. (2002). Physical evidence of police officer stress. *Policing: An International Journal of Police Strategies & Management* 25(2)**,** 399-420. doi: 10.1108/13639510210429437.

Appelhans, B.M., and Luecken, L.J. (2006). Heart Rate Variability as an Index of Regulated Emotional Responding. *Review of General Psychology* 10(3)**,** 229-240. doi: 10.1037/1089-2680.10.3.229.

Ayuso-Moreno, R., Fuentes-García, J.P., Collado-Mateo, D., and Villafaina, S. (2020). Heart rate variability and pre-competitive anxiety according to the demanding level of the match in female soccer athletes. *Physiology & Behavior* 222**,** 112926. doi: 10.1016/j.physbeh.2020.112926.

Baevsky, R.M. (2009). Methodical recommendations use kardivar system for determination of the stress level and estimation of the body adaptability standards of measurements and physiological interpretation.

Baldwin, S., Bennell, C., Andersen, J.P., Semple, T., and Jenkins, B. (2019). Stress-Activity Mapping: Physiological Responses During General Duty Police Encounters. *Frontiers in Psychology* 10(2216). doi: 10.3389/fpsyg.2019.02216.

Berntson, G.G., and Cacioppo, J.T. (2004). "Heart Rate Variability: Stress and Psychiatric Conditions," in *Dynamic Electrocardiography*. Blackwell Publishing), 57-64.

Berntson, G.G., Thomas Bigger, J., Eckberg, D.L., Grossman, P., Kaufmann, P.G., Malik, M., et al. (1997). Heart rate variability: Origins, methods, and interpretive caveats. *Psychophysiology* 34(6)**,** 623-648. doi: 10.1111/j.1469-8986.1997.tb02140.x.

Brisinda, D., Venuti, A., Cataldi, C., Efremov, K., Iantorno, E., and Fenici, R. (2015). Real-time Imaging of Stress-induced Cardiac Autonomic Adaptation During Realistic Force-on-force Police Scenarios. *Journal of Police and Criminal Psychology* 30(2)**,** 71-86. doi: 10.1007/s11896-014-9142-5.

Castaldo, R., Melillo, P., Bracale, U., Caserta, M., Triassi, M., and Pecchia, L. (2015). Acute mental stress assessment via short term HRV analysis in healthy adults: A systematic review with meta-analysis. *Biomedical Signal Processing and Control* 18**,** 370-377. doi: 10.1016/j.bspc.2015.02.012.

Fenici, R., Brisinda, D., and Sorbo, A.R. (2011). "Methods for Real-Time Assessment of Operational Stress during Realistic Police Tactical Training," in *Handbook of police psychology,* ed. J. Kitaeff. (Florence, US: Routledge), 295.

Giessing, L., Frenkel, M.O., Zinner, C., Rummel, J., Nieuwenhuys, A., Kasperk, C., et al. (2019). Effects of Coping-Related Traits and Psychophysiological Stress Responses on Police Recruits' Shooting Behavior in Reality-Based Scenarios. *Frontiers in Psychology* 10**,** 1523-1523. doi: 10.3389/fpsyg.2019.01523.

Giuseppe, G., Antonio, B., Luigi Isaia, L., Nicola, M., Marco, P., Mario, N., et al. (2021). HRV in Active-Duty Special Forces and Public Order Military Personnel. *Sustainability (Basel, Switzerland)* 13(3867)**,** 3867. doi: 10.3390/su13073867.

Haller, J., Raczkevy-Deak, G., Gyimesine, K.P., Szakmary, A., Farkas, I., and Vegh, J. (2014). Cardiac autonomic functions and the emergence of violence in a highly realistic model of

social conflict in humans. *Frontiers in behavioral neuroscience* 8**,** 364. doi: 10.3389/fnbeh.2014.00364.

James, L., Goldstein Michael, S., Lecy, P., and Mase, S. (2020). Testing the impact of physiological stress response on police performance during critical job tasks. *Policing: An International Journal* ahead-of-print(ahead-of-print). doi: 10.1108/PIJPSM-04-2020-0060.

Korotkov, K.G. (2017). Gender Differences in the Activity of the Autonomic Nervous Systems of Healthy and Hypertensive Patients in Russia. *Journal of Applied Biotechnology & Bioengineering* 3(6). doi: 10.15406/jabb.2017.03.00084.

Kubios Oy (2021). *HRV in evaluating ANS function* [Online]. Kuopio, Finland: Kubios Oy. Available: https://www.kubios.com/hrv-ans-function/ [Accessed].

Laborde, S., Mosley, E., and Thayer, J.F. (2017). Heart Rate Variability and Cardiac Vagal Tone in Psychophysiological Research – Recommendations for Experiment Planning, Data Analysis, and Data Reporting. *Frontiers in Psychology* 08. doi: 10.3389/fpsyg.2017.00213.

Lipponen, J.A., and Tarvainen, M.P. (2019). A robust algorithm for heart rate variability time series artefact correction using novel beat classification. *Journal of Medical Engineering & Technology* 43(3)**,** 173-181. doi: 10.1080/03091902.2019.1640306.

Lundell, R.V., Tuominen, L., Ojanen, T., Parkkola, K., and Räisänen-Sokolowski, A. (2021). Diving Responses in Experienced Rebreather Divers: Short-Term Heart Rate Variability in Cold Water Diving. *Frontiers in Physiology* 12(457). doi: 10.3389/fphys.2021.649319.

Malik, M., Bigger, J.T., Camm, A.J., Kleiger, R.E., Malliani, A., Moss, A.J., et al. (1996). Heart rate variability : Standards of measurement, physiological interpretation, and clinical use. *European heart journal* 17(3)**,** 354-381. doi: 10.1093/oxfordjournals.eurheartj.a014868.

Munoz, M.L., van Roon, A., Riese, H., Thio, C., Oostenbroek, E., Westrik, I., et al. (2015). Validity of (Ultra-)Short Recordings for Heart Rate Variability Measurements. *PLoS ONE* 10(9)**,** e0138921. doi: 10.1371/journal.pone.0138921.

Rajendra, A.U., Paul, J.K., Kannathal, N., Lim, C.M., and Suri, J.S. (2006). Heart rate variability: a review. *Medical & Biological Engineering & Computing* 44(12)**,** 1031-1051. doi: 10.1007/s11517-006-0119-0.

Smith, A.-L., Owen, H., and Reynolds, K. (2013). Heart rate variability indices for very short-term (30 beat) analysis. Part 1: survey and toolbox. *Journal of Clinical Monitoring and Computing* 27(5)**,** 569-576. doi: 10.1007/s10877-013-9471-4.

Tarvainen, M.P., Lipponen, J.A., Niskanen, J.-P., and Ranta-aho, P.O. (2016). "Kubios HRV (ver. 3.0) User's Guide". (Kuopio, Finland: Kubios Oy).

Tarvainen, M.P., Lipponen, J.A., Niskanen, J.-P., and Ranta-aho, P.O. (2020). "Kubios HRV Software User's Guide". (Kuopio, Finland: Kubios Oy).

Thayer, J.F., Ahs, F., Fredrikson, M., Sollers, J.J., and Wager, T.D. (2012). A meta-analysis of heart rate variability and neuroimaging studies: implications for heart rate variability as a marker of stress and health. *Neuroscience and biobehavioral reviews* 36(2)**,** 747.
