# Supplementary material for: A Reasonable Officer: Examining the Relationships Among Stress, Training, and Performance in a Highly Realistic Lethal Force Scenario
Source: Front Psychol. 2022 Jan 17;12:759132. doi: 10.3389/fpsyg.2021.759132 (PMC8803048; doi:10.3389/fpsyg.2021.759132)
Supplement: SUPPLEMENTARY MATERIAL INDEX — https://doi.org/10.17605/OSF.IO/PKJNV. [file Data_Sheet_1.zip › Supplementary Material C.pdf]

Supplementary Material C - Self-Reported Perceptual and Cognitive Distortions Questionnaire

During the scenario, I experienced:	Not at all	Very little	Somewhat	To a great extent
diminished sound (i.e., inability to hear very loud sounds I would ordinarily obviously hear, such as gunshots, shouting, etc)				
intensified sounds				
heightened visual clarity				
tunnel vision (i.e., loss or narrowing of peripheral vision)				
automatic pilot (i.e., I responded with little or no conscious thought)				
slow motion time (i.e., time slowed down)				
fast motion time (i.e., time sped up)				
temporary paralysis (i.e., froze)				
dissociation (i.e., a sense of detachment or unreality)				
intrusive distracting thoughts (i.e., thoughts not immediately relevant to the tactical situation, often including thoughts about loved ones or other personal matters)				
Post-scenario, I experienced:	Not at all	Very little	Somewhat	To a great extent
memory loss for part of the event				
memory loss for some of my own behavior				
memory distortions (i.e., I saw, heard, or experienced something that didn't really happen or happened very differently)				
"flashbulb" memories, where I have a series of vivid images burned into memory, with the rest of the event somewhat fuzzy or missing.				

(Adapted from Artwohl, 2008)

References

Artwohl, A. (2008). Perceptual and Memory Distortion During Officer-Involved Shootings. *FBI Law Enforcement Bulletin* 71(10)**,** 18-24.
