# Supplementary material for: A Reasonable Officer: Examining the Relationships Among Stress, Training, and Performance in a Highly Realistic Lethal Force Scenario
Source: Front Psychol. 2022 Jan 17;12:759132. doi: 10.3389/fpsyg.2021.759132 (PMC8803048; doi:10.3389/fpsyg.2021.759132)
Supplement: SUPPLEMENTARY MATERIAL INDEX — https://doi.org/10.17605/OSF.IO/PKJNV. [file Data_Sheet_1.zip › Supplementary Material D.pdf]

Supplementary Material D - Agency Training and Assessment of Level of Training

Prior to becoming an operational police officer with the agency, all recruits must attend an extensive 26-week basic training program where they receive foundational skills. This includes approximately 3 ½ weeks of use of force (UoF) training, involving 64 hours of firearms safety, law and policy, marksmanship, and decision-making, as well as 75 hours on police defensive tactics, including skills acquisition, principles of UoF,[1] and scenario-based training (SBT).[2] Additionally, de-escalation skills (e.g., verbal and non-verbal communication) are taught throughout training and are then applied during a full day of scenarios in which the clients are in various states of emotional distress. This is followed by 26-weeks of on-the-job learning under the supervision of a field training officer. Once in the field, officers complete an average of approximately 40 hours of training per year.[3]

Officers' training records and the training information captured in the demographics form were used to identify and assess their level of in-service training. To assess the level of training

[1] In Canada, guiding principles for the use of force also come from the national use of force framework or related models (Canadian Association of Chiefs of Police, 2000). These models are visual aids that were developed for training purposes, to assist officers in making appropriate decisions during interactions with the public and help officers to better articulate their actions after-the-fact (Hoffman et al., 2004). The model and supporting training focus on the totality of the circumstances that should enter into an officer's continuous risk assessment (i.e., officer perceptions, situational factors, tactical consideration and the subject's behavior). The totality of these factors assists officers in determining which intervention option (e.g., communication, intermediate weapon, lethal force) is most appropriate to control the situation. Within the organizational context, the model is fundamental for assessing use of force performance in operational and training settings.

[2] SBT typically involves officers being equipped with inert intervention options (e.g., rubber baton, inert OC spray, and taser), as well as a firearm loaded with non-lethal rounds (i.e., paint-based marking cartridges). The candidate is run through a scenario facilitated by an instructor. The role-player plays the role of a suspect who either escalates or de-escalates the situation based on the response of the officer. Officer performance is assessed throughout the scenario and sometimes in their post-incident articulation of their actions.

[3] In the US, the average length of police academy training is approximately 21 weeks (i.e., 840 hours), of which around 171 hours (20%) are dedicated to the use of force (Reaves, 2016). This is typically followed by 13 weeks (i.e., 520 hours) of on-the-job learning under the supervision of a field training officer (Reaves, 2016). Once on active duty, officers receive on average less than one week (i.e., 35 hours) of training per year (Reaves, 2010).

for non-elite and advanced participants, the sum of the following 12 equally weighted operational skills courses was calculated: (1) crisis intervention and de-escalation, (2) first aid and/or CPR instructor, (3) basic trauma equipment instructor, (4) conducted energy weapon, (5) extended range impact weapon, (6) VIP close protection, (7) tactical support group (i.e., crowd control), (8-9) advanced firearms, and (10-12), active threat training (three separate courses).

To account for the recency and frequency of training experience, courses were only included if they were completed within the last 5 years and/or were completed more than once; with the exception of (2) and (3), which were based on whether participants were ever instructors. Years of police service was also assessed to account for annual firearm qualifications and triennial operational skills maintenance training (i.e., OC spray, baton, carotid control, first aid, CPR and SBT).

References

Canadian Association of Chiefs of Police (2000). "A National Use of Force Framework". (Ottawa, Canada: Canadian Association of Chiefs of Police).

Hoffman, R., Lawrence, C., and Brown, G. (2004). Canada’s National Use-of-Force Framework for Police Officers. *The Police Chief* 71(10).

Reaves, B.A. (2010). "Local Police Departments, 2007". (Washington, DC).

Reaves, B.A. (2016). "State and Local Law Enforcement Training Academies, 2013". (Washington, DC).
