# Supplementary material for: A Reasonable Officer: Examining the Relationships Among Stress, Training, and Performance in a Highly Realistic Lethal Force Scenario
Source: Front Psychol. 2022 Jan 17;12:759132. doi: 10.3389/fpsyg.2021.759132 (PMC8803048; doi:10.3389/fpsyg.2021.759132)
Supplement: SUPPLEMENTARY MATERIAL INDEX — https://doi.org/10.17605/OSF.IO/PKJNV. [file Data_Sheet_1.zip › Supplementary Material E.pdf]

Supplementary Material E - Deadly Force Judgment and Decision-Making (DFJDM), Tactical Social Interaction (TSI), and Crisis Intervention Team (CIT; adapted from Vila et al., 2018)

1. Pre-Planning…seeking accurate information about the situation before arrival (weight: 4)
2. Pre-Planning…seeking accurate information about the person in crisis before arrival (weight: 4)
3. Assess…recognizing weapons of opportunity in the environment (e.g. machete, baseball bat, sledgehammer, axe, or recognizes knife before drawn) (weight: 4)
4. Assess…observing details of the environment before the encounter starts (e.g. note exit strategy, good scan of back room) (weight: 4)
5. Tactics…removing non-involved participants from the encounter (e.g. bystanders, family members) (weight: 4)
6. Officer Behavior...Asking questions that are relevant to the mission... (e.g. for breach, assault, location of victim) (weight: 6)
7. Observe and Assess…the officer is able to identify the suspect's mental or physical health (e.g., understands threat to self-harm) (weight: 3)
8. Interacting with the Person in Crisis/Officer Behavior…being able to actively listen to the person in crisis during the encounter (e.g., paraphrasing, nonverbal cues which show understanding, verbal affirmations) (weight: 4)
9. Interacting with the Person in Crisis/Officer Behavior…being able to show empathy to the person in crisis (e.g., being aware of, being sensitive to, and vicariously experiencing the feelings, thoughts, and experience) (weight: 4)
10. Interacting with the Person in Crisis/Officer Behavior…having the ability to de-escalate a situation (calm the person in crisis down) (weight: 4)
11. Officer Behavior - Offering to help the civilian…(e.g., I want to help, I'm here to help) (weight: 6)
12. Interacting with the Person in Crisis/Officer Behavior…demonstrating concern for the person in crisis's safety (weight: 4)
13. Interacting with the Person in Crisis/Officer Behavior…demonstrating patience with the person in crisis (weight: 4)
14. Officer Behavior…the officer makes timely decisions regarding pre-assault indicators (e.g., immediately drawing firearm when knife is pulled) (weight: 5)
15. Observe and Assess…the officer selects reasonable force options (weight: 5)
16. Adapt…the officer recognized the need to transition to other force options (N/A if there was no need to transition - e.g., firearm) (weight: 5)
17. Tactics…being proficient with standard equipment (weight: 4)
18. Officer Behavior…the officer applies deadly force rules of engagement (laws and policies) in a combat situation (weight: 4)
19. Training and Wellness…the officer can shoot proficiently under combat conditions (weight: 5)
20. Training and Wellness…the officer can tactically load and reload weapons under combat conditions (N/A if not applicable) (weight: 5)

21. Training and Wellness…whether the officer can effectively clear malfunctions under combat conditions (N/A if not applicable) (weight: 4)
22. Self-Control/Officer Characteristics…practicing self-control techniques during the encounter (e.g. deep breathing) (weight: 4)
23. Tactics…the officer manages their perceptual narrowing during a deadly encounter (e.g., stuck in loop, stunned, inappropriate behaviour) (weight: 4)
24. Adapt/Repair - Recognizing when their actions are not appropriate and modifying them...(N/A if not applicable) (weight: 7)
25. Tactics…being proficient with control techniques (e.g., tactical disadvantage and handcuffing techniques) (weight: 3)
26. Officer Behavior - Taking action to improve civilian's conditions…(e.g., providing medical attention) (weight: 6)
27. Tactics…calling for back-up when appropriate (e.g., when knife is pulled and shots are fired) (weight: 4)
28. Officer Behavior…the officer is able to communicate key information to [dispatch] (weight: 4)
29. Observe and Assess…when possible, the officer assesses the situation fully before acting (weight: 5)
30. Observe and Assess…the officer overestimates their ability to read a situation (weight: -5)
31. Officer Behavior…the officer used an appropriate level of aggressiveness (weight: 4)
32. Officer Behavior…the officer used an appropriate level of assertiveness (weight: 4)
33. Officer Behavior…the officer maintains control of the encounter until it is resolved (weight: 5)
34. Tactics…the officer gives relevant and meaningful commands (weight: 5)
35. Tactics…the officer knows their position relative to bystanders (e.g., maintains subject in periphery, does not turn back for extended period when in close proximity) (weight: 3)
36. Tactics…the officer makes full use of available cover and concealment (weight: 5)
37. Tactics…the officer makes partial use of available cover and concealment (weight: 2)
38. Tactics…the officer optimizes the distance between him or herself and the identified threat (e.g., back of room, doorway) (weight: 4)
39. Officer Behavior - Maintaining a position of tactical advantage... (weight: 7)

Note. For coding purposes, any indicators that were not completely objective (e.g., "the officer makes timely decisions regarding pre-assault indicators") were accompanied with examples (e.g., immediately drawing firearm when knife is pulled; Vila et al., 2018).

References

Vila, B., James, S., and James, L. (2018). How police officers perform in encounters with the public: Measuring what matters at the individual level. *Policing: An International Journal* 41(2)**,** 215-232. doi: 10.1108/PIJPSM-11-2016-0166.
