# Supplementary material for: A Reasonable Officer: Examining the Relationships Among Stress, Training, and Performance in a Highly Realistic Lethal Force Scenario
Source: Front Psychol. 2022 Jan 17;12:759132. doi: 10.3389/fpsyg.2021.759132 (PMC8803048; doi:10.3389/fpsyg.2021.759132)
Supplement: SUPPLEMENTARY MATERIAL INDEX — https://doi.org/10.17605/OSF.IO/PKJNV. [file Data_Sheet_1.zip › Supplementary Material F.pdf]

Supplementary Material F - Agency Performance Metrics (adapted)

Professionalism, law, & policy (3 measures)

- Advises subject of arrest unless situational factors dictate otherwise.
- Makes timely decisions in accordance with the law and policy.
- Interaction is respectful and appropriate.

Skills & techniques (5 measures)

- Precise demonstration of techniques and skills.
- Fluid and subconscious movement.
- Controls the subject effectively.
- Manipulates intervention option(s) effectively.
- Transitions smoothly to other techniques and/or intervention option(s).

Tactics and officer safety (28 measures)

- Uses new strategies.
- Recognizes subtleties in threat cues, environment and body language and responds appropriately.
- Demonstrates fast and fluid actions.
- Adapts to transitions in behaviour rapidly.
- Voices commands precisely and directly.
- Advises subject what they want them to do (versus not do).
- Applies the appropriate tactical considerations in response to the applicable threat cue(s) in a timely manner.
    - Cover & concealment
    - Threat cues
    - Time & distance
    - 1+1 principle
    - Verbalization
    - De-escalation
    - Survival mentality
    - Maintains control of the situation
- Applies the 4 Cs:
    - Check your environment
    - Condition (self)
    - Condition (subject)
    - Communication
    - Combat breathing
- Applies DARCS
    - Double-lock
    - Reason for arrest
    - Charter rights (i.e., right to retain and instruct a lawyer without delay)
    - Caution (i.e., not obliged to say anything, but anything you do say, can be given in evidence)
    - Search
- Applies ALPS for search
    - Ask

- Look
- Pat
- Squeeze

Medical response (8 measures)

- Simulated or verbalized basic first aid (e.g., wound pressure)
- Assessed for penetrating chest injury by exposing wound and/or conducting a closed claw rake of the chest/abdominal injury site(s)
- Immediately occluded/covered the penetrating open chest wound (e.g., with hand)
- Thoroughly raked (close claw technique) all sides of the chest (front and back) to assess for additional injury sites (i.e., exit wounds)
- Simulated or verbalized application of a chest seal (vented chest seal preferred)
- Placed casualty in the recovery position (injured side down for unconscious casualty) or a position of comfort (conscious casualty)
- Assessed airway, and quality of breathing frequently
- Verbalized the need to transport the casualty to EMS ASAP via radio communication
