# Supplementary material for: A Reasonable Officer: Examining the Relationships Among Stress, Training, and Performance in a Highly Realistic Lethal Force Scenario
Source: Front Psychol. 2022 Jan 17;12:759132. doi: 10.3389/fpsyg.2021.759132 (PMC8803048; doi:10.3389/fpsyg.2021.759132)
Supplement: SUPPLEMENTARY MATERIAL INDEX — https://doi.org/10.17605/OSF.IO/PKJNV. [file Data_Sheet_1.zip › Supplementary Material G.pdf]

Supplementary Material G - Scenario Training Assessment and Review (STAR) Scale (adapted from Wollert et al., 2011)

For the current study, categories and descriptors were slightly adapted to reflect Canadian law, as well as agency terminology, training, policy, procedures, and the national use of force framework. Additionally, articulation/after action review performance was not assessed as part of this study and was instead replaced with a medical attention performance category.

1. **Situational awareness:** Awareness of surroundings, threats, risks and understanding of how information, events, and actions will impact goals and objectives, both now and in the near future.
2. **Threat/risk assessment and identification:** Threats, non-threats and risks are accounted for, properly prioritized, effectively communicated, and appropriate response is efficiently planned.
3. **Initial response:** Strategy to properly respond to threats, risks and situation including position of advantage, tactics, or other corrective actions.
4. **Scene control after the initial response:** Strategy to maintain control of the situation including evidence, crime scene, threats, victims, and witnesses.
5. **Use-of-force:** Application of appropriate/timely force options consistent with Charter of Rights, Criminal Code and case law.
6. **Arrest procedures:** Initiation of correct procedures during an arrest including position of disadvantage, handcuffing, rights, caution and search.
7. **Communication:** Information exchange between entities through correct/timely verbal commands, non-verbal behaviors, and de-escalation.
8. **Medical:** Application of skills necessary to help sustain life and minimize the consequences of injury until advanced medical help is available (e.g., Desirable = Tactical first aid and trauma equipment; Acceptable = Basic First Aid; Least desirable = wound pressure only; Not acceptable = no medical attention).

Rating & description:

0. Not acceptable: Critical errors. Performance is not consistent with legal standard, creates serious risk, or did not perform.
1. Least desirable: Non-critical errors. Performance is generally acceptable but creates identifiable risk.
2. Acceptable: Working level. Performance is consistent with training but not the most effective method (e.g., action, tactic).
3. Desirable: Demonstrates sound and effective performance.

Not applicable: Does not apply or is not observable.

References

Wollert, T., Driskell, J.E., and Quail, J. (2011). Stress exposure training guidelines: Instructor guide to reality-based training.
