# Supplementary material for: A Reasonable Officer: Examining the Relationships Among Stress, Training, and Performance in a Highly Realistic Lethal Force Scenario
Source: Front Psychol. 2022 Jan 17;12:759132. doi: 10.3389/fpsyg.2021.759132 (PMC8803048; doi:10.3389/fpsyg.2021.759132)
Supplement: SUPPLEMENTARY MATERIAL INDEX — https://doi.org/10.17605/OSF.IO/PKJNV. [file Data_Sheet_1.zip › Supplementary Material H.pdf]

Supplementary Material H - Descriptive Statistics and Correlation Matrix for Performance Metrics (*N* =122)

	M	*SD*	*Min*	*Max*	Agency performance metric	STAR Scale
DFJDM, TSI, and CIT metric	65.77	17.53	21.95	100.00	.631***	.667***
Agency performance metric	61.86	11.73	30.23	88.64		.615***
STAR Scale	50.29	18.60	12.50	91.67		
Overall performance rating	59.31	13.96	24.99	93.43		

Note. ***indicates $p < 0.001$.
