# Supplementary material for: A Reasonable Officer: Examining the Relationships Among Stress, Training, and Performance in a Highly Realistic Lethal Force Scenario
Source: Front Psychol. 2022 Jan 17;12:759132. doi: 10.3389/fpsyg.2021.759132 (PMC8803048; doi:10.3389/fpsyg.2021.759132)
Supplement: SUPPLEMENTARY MATERIAL INDEX — https://doi.org/10.17605/OSF.IO/PKJNV. [file Data_Sheet_1.zip › Supplementary Material I.pdf]

Supplementary Material I - Performance by Level of Training

Level of Training	Overall performance rating		DFJDM, TSI, and CIT metric		Agency performance metric		STAR scale	
	M	*SD*	*M*	*SD*	*M*	*SD*	*M*	*SD*
Elite (level 2)	73.57	9.92	77.72	15.42	74.84	7.37	68.15	13.44
Elite (level 1)	69.84	10.90	76.53	15.17	68.40	9.11	64.58	15.81
Advanced	59.47	8.32	63.39	13.60	65.72	7.38	49.31	15.87
Intermediate (level 3)	56.18	14.29	61.93	20.02	61.18	9.99	45.42	19.19
Intermediate (level 2)	55.60	10.89	64.09	17.01	58.87	8.79	43.83	12.74
Intermediate (level 1)	57.89	11.17	66.56	14.00	58.56	11.24	48.54	16.57
Novice/basic (level 2)	50.76	17.15	56.07	20.40	53.31	13.72	42.89	20.40
Novice/basic (level 1)	50.24	11.92	55.64	12.59	56.82	10.78	38.28	19.79
Total	59.31	13.96	65.77	17.53	61.86	11.73	50.29	18.60

Note. N = 122.
