# Supplementary material for: A Reasonable Officer: Examining the Relationships Among Stress, Training, and Performance in a Highly Realistic Lethal Force Scenario
Source: Front Psychol. 2022 Jan 17;12:759132. doi: 10.3389/fpsyg.2021.759132 (PMC8803048; doi:10.3389/fpsyg.2021.759132)
Supplement: SUPPLEMENTARY MATERIAL INDEX — https://doi.org/10.17605/OSF.IO/PKJNV. [file Data_Sheet_1.zip › Supplementary Material J.pdf]

Supplementary Material J - Type of Lethal Force Error by Level of Training

	Type of lethal force error							
	None		Decision-making error		Mistake of fact error		Both	
Level of Training	*n*	%	*n*	%	*n*	%	*n*	%
Elite (level 2)	12	85.7%	1	7.1%	1	7.1%	0	0.0%
Elite (level 1)	10	62.5%	1	6.3%	4	25.0%	1	6.3%
Advanced	5	41.7%	3	25.0%	2	16.7%	2	16.7%
Intermediate (level 3)	5	50.0%	1	10.0%	3	30.0%	1	10.0%
Intermediate (level 2)	18	72.0%	2	8.0%	5	20.0%	0	0.0%
Intermediate (level 1)	18	90.0%	0	0.0%	2	10.0%	0	0.0%
Novice/basic (level 2)	13	76.5%	0	0.0%	3	17.6%	1	5.9%
Novice/basic (level 1)	7	87.5%	1	12.5%	0	0.0%	0	0.0%
Total	88	72.1%	9	7.4%	20	16.4%	5	4.1%

Note. $N = 122$.
