# Supplementary material for: A Reasonable Officer: Examining the Relationships Among Stress, Training, and Performance in a Highly Realistic Lethal Force Scenario
Source: Front Psychol. 2022 Jan 17;12:759132. doi: 10.3389/fpsyg.2021.759132 (PMC8803048; doi:10.3389/fpsyg.2021.759132)
Supplement: SUPPLEMENTARY MATERIAL INDEX — https://doi.org/10.17605/OSF.IO/PKJNV. [file Data_Sheet_1.zip › Supplementary Material K.pdf]

Supplementary Material K - Exploratory Analysis of Behavioural Predictors of Performance

To examine which individual behaviours were most associated with overall scores on the performance metrics, a series of non-parametric correlations were calculated. Given the exploratory nature of this analysis, Bonferroni corrections were not applied (Armstrong, 2014). Table 9 presents all behaviours with a large correlation (r_s >=.5) with at least one of the performance metrics. The table also includes correlations with level of training to assess whether these behaviours were associated with agency training.

Several trends emerged from the data, with results indicating that assessing the situation, recognizing threat cues, maintaining tactical advantage (i.e., time, distance, cover, concealment), and competence with intervention options, were all highly associated with performance in the scenario and moderately associated with level of training (r_s > .27). Overall verbal de-escalation and relevant de-escalation behaviours (e.g., demonstrating patience, offering help, appropriate level of aggressiveness) were also highly associated with overall performance; however, they demonstrated small (r_s < .14) non-significant associations with level of current police training (p >.05).

Table 9

Individual Performance Items Associated (r_s >=.5) with Performance Metrics and Level of Training

	Overall performance rating	DFJDM, TSI, and CIT	Agency performance metrics	STAR scale	Level of training
Observe and Assess…when possible, the officer assesses the situation fully before acting.	.739***	.810***	.445***	.597***	.290**

Recognizes subtleties in threat cues, environment and body language and responds appropriately.	.693***	.569***	.559***	.657***	.272**
Observe and Assess…the officer overestimates their ability to read a situation.	-.625***	-.715***	-.381***	-.474***	-.345***
Officer Behavior - Maintaining a position of tactical advantage...	.610***	.659***	.433***	.476***	.284**
Adapt/Repair - Recognizing when their actions are not appropriate and modifying them...(*n* = 93)	.609***	.572***	.426***	.588***	0.199
Threat cues	.573***	.464***	.461***	.542***	.275**
Cover & concealment	.570***	.586***	.439***	.452***	.419***
Tactics…the officer manages their perceptual narrowing during a deadly encounter.	.566***	.538***	.362***	.503***	.191*
Tactics…the officer optimizes the distance between him or herself and the identified threat.	.565***	.625***	.415***	.405***	.382***
Time & distance	.561***	.621***	.428***	.400***	.381***
Interacting with the Person in Crisis/Officer Behavior…demonstrating patience with the person in crisis	.499***	.663***	.245**	.337***	0.133
Officer Behavior…the officer used an appropriate level of aggressiveness.	.499***	.513***	.230*	.468***	0.112
Transitions smoothly to other techniques and/or intervention option(s). (*n* = 71)	.464***	.347**	.519***	.421***	.349**
Tactics…the officer makes full use of available cover and concealment.	.443***	.516***	.254**	.349***	.455***
De-escalation	.412***	.578***	0.158	.252**	0.027
Precise demonstration of techniques and skills. (*n* = 121)	.396***	.300***	.502***	.316***	.323***
Interacting with the Person in Crisis/Officer Behavior…having the ability to de-escalate a situation (calm the person in crisis down)	.394**	.562***	0.167	.216*	-0.006

Officer Behavior - Offering to help the civilian…	$.352^{**}$	$.542^{**}$	0.138	0.168	0.086

Note. *indicates $p < 0.05$. **indicates $p < 0.01$. ***indicates $p < 0.001$.

References

Armstrong, R.A. (2014). When to use the Bonferroni correction. *Ophthalmic and Physiological Optics* 34(5)**,** 502-508. doi: 10.1111/opo.12131.
