# Supplementary material for: A Reasonable Officer: Examining the Relationships Among Stress, Training, and Performance in a Highly Realistic Lethal Force Scenario
Source: Front Psychol. 2022 Jan 17;12:759132. doi: 10.3389/fpsyg.2021.759132 (PMC8803048; doi:10.3389/fpsyg.2021.759132)
Supplement: SUPPLEMENTARY MATERIAL INDEX — https://doi.org/10.17605/OSF.IO/PKJNV. [file Data_Sheet_1.zip › Supplementary Material L.pdf]

Supplementary Material L - Context on the Frequency of Police Use of Force and Training

For context, estimates consistently identify that approximately 0.1% of police occurrences in Canada involve UoF (Hall and Votova, 2013; Baldwin et al., 2020; Walker and Bennell, 2021). Additionally, over a five-year period (2015-2019), public databases of fatal police encounters in Canada, identified 136 individuals fatally shot by police (Singh, 2020) and 7 police officers were criminally killed in the line of duty (Memorial Ribbon Society, 2021). In comparison, in the United States (US), similar UoF estimates have recently been reported (Bozeman et al., 2018), although others have estimated that 1.7% of police-public interactions in the US involve the use or threat of force (Hickman et al., 2008). Additionally, from 2015-2019 in the US, public databases recorded just over 4,900 individuals fatally shot by law enforcement officers (Tate et al., 2021) and 257 officers were criminally killed (Federal Bureau of Investigations, 2020). Based on these estimates, with a population approximately 9-times larger than Canada, the US demonstrated 36 times more individuals fatally shot by police and 37 times more officers criminally killed in the line of duty.

While LEAs train officers for critical incidents, training time is often restricted due to resource and budgetary constraints (Bennell et al., 2020; Rojek et al., 2020). Consequently, officers receive considerably less training relative to other professions that require high-stakes decision-making (e.g., doctors, nurses, surgeons, airline pilots; Di Nota and Huhta, 2019). For example, in the United States, the average reported length of police academy training is approximately 21 weeks (i.e., 840 hours), of which around 171 hours (20%) are dedicated to the use of force (Reaves, 2016). This is typically followed by 13 weeks (i.e., 520 hours) of on-the-job learning under the supervision of a field training officer (Reaves, 2016). Once on active duty, officers receive on average less than one week (i.e., 35 hours) of training per year (Reaves,

2010). In Canada, similar training requirements exist. Additionally, the minimum education standards to apply for law enforcement in Canada are normally a high school diploma or equivalent. In contrast, registered nurses are typically required to complete a four-year university nursing program to become a generalist (National Nursing Assessment Service, 2021) and teachers generally require a minimum three-year postsecondary degree and a two-year teacher education program (Ontario College of Teachers, 2021).

Thus, officers often have limited opportunity in training and the field to use various knowledge, skills, and abilities (KSAs), such as the use of force (UoF; e.g., Hall and Votova, 2013), which can impact retention (Di Nota and Huhta, 2019; O’Neill et al., 2019).

References

Baldwin, S., Blaskovits, B., Widdershoven, N., and Marchant, S. (2020). "2010 to 2019 Police Intervention Options Report". (Ottawa: Royal Canadian Mounted Police).

Bennell, C., Blaskovits, B., Jenkins, B., Semple, T., Khanizadeh, A.-J., Brown Andrew, S., et al. (2020). Promising practices for de-escalation and use-of-force training in the police setting: a narrative review. *Policing: An International Journal* ahead-of-print(ahead-of-print). doi: 10.1108/PIJPSM-06-2020-0092.

Bozeman, W.P., Stopyra, J.P., Klinger, D.A., Martin, B.P., Graham, D.D., Johnson, J.C.I., et al. (2018). Injuries associated with police use of force. *Journal of Trauma and Acute Care Surgery* 84(3)**,** 466-472. doi: 10.1097/ta.0000000000001783.

Di Nota, P.M., and Huhta, J.-M. (2019). Complex Motor Learning and Police Training: Applied, Cognitive, and Clinical Perspectives. *Frontiers in Psychology* 10(1797). doi: 10.3389/fpsyg.2019.01797.

Hall, C.A., and Votova, K. (2013). "Prospective analysis of police use of force in four Canadian cities: nature of events and their outcomes". (Ottawa, Canada: Defence Research and Development Canada).

Memorial Ribbon Society (2021). *The Police & Peace Officers' Memorial Ribbon Honour Roll remembers all Police and Peace Officers in Canada who have died while serving their communities* [Online]. Memorial Ribbon Society. Available: https://www.memorialribbon.org/honour-roll/ [Accessed June 26 2021].

National Nursing Assessment Service (2021). *RN, RPN and LPN requirements in Canada - National Nursing Assessment Service* [Online]. Available: https://www.nnas.ca/nursing-requirements-in-canada/ [Accessed].

O'Neill, J., O'Neill, D.A., Weed, K., Hartman, M.E., Spence, W., and Lewinski, W.J. (2019). Police Academy Training, Performance, and Learning. *Behavior Analysis in Practice* 12(2)**,** 353-372. doi: 10.1007/s40617-018-00317-2.

Ontario College of Teachers (2021). *Requirements | Ontario College of Teachers* [Online]. Available: https://www.oct.ca/becoming-a-teacher/requirements [Accessed].

Reaves, B.A. (2010). "Local Police Departments, 2007". (Washington, DC).

Reaves, B.A. (2016). "State and Local Law Enforcement Training Academies, 2013". (Washington, DC).

Rojek, J., Grieco, J., Meade, B., and Parsons, D. (2020). "National Survey on Officer Safety Training: Findings and Implications". (Washington, DC).

Singh, I. (2020). 2020 already a particularly deadly year for people killed in police encounters, CBC research shows. *CBC News*.

Walker, T., and Bennell, C. (2021). "Use of Force by Canadian Police Officers", in: *Canadian Police Chief Magazine.* (Kanata, Ontario: Naylor).
